# Synthesis of trifunctional indole-imine-based Ag NPs as a molecular probe for selective colorimetric detection of Cd(ii), photo-catalytic and antimicrobial agent

**DOI:** 10.1039/d5ra07704d

**Published:** 2026-02-18

**Authors:** Muhammad Kashif, Abdul Rauf Raza, Shoaib Akhtar, Khaled Fahmy Fawy, Muhammad Sher, Umar Nishan, Muhammad Imran Irfan, Azhar Abbas

**Affiliations:** a Institute of Chemistry, University of Sargodha Ibn e Sina Block Sargodha 40100 Pakistan rauf.raza@uos.edu.pk azhar.ramzan@uos.edu.pk +92301148784 +923339793986; b Central Labs, King Khalid University AlQura’a, Abha P. O. Box 960, 61413 Saudi Arabia; c Department of Chemistry, Kohat University of Science and Technology Kohat KP 26000 Pakistan; d Department of Chemistry, Government Ambala Muslim College Sargodha 40100 Pakistan

## Abstract

In the present study, a novel *N*-(4′-methylphenyl) (4,6-dimethoxy-2,3-diphenyl-1*H*-indol-7-yl)methanimine 6 (indole imine 6) was synthesized in good yield and was characterized through FT-IR, UV-Vis, GC-MS, ^1^H/^13^C NMR, and single crystal XRD techniques. The resulting indole-imine Schiff base serves a dual role as both a reducing and stabilizing agent for the single-pot synthesis of AgNPs-6 in a non-aqueous medium. These AgNPs-6 were then characterized using UV-Vis, FT-IR, Scanning-Electron-Microscopy-Energy-Dispersive-X-Rays (SEM-EDX), Dynamic Light Scattering (DLS), and Zeta Potential measurement. The synthesized AgNPs-6 demonstrated remarkable trifunctionality: it operated as a sensitive colorimetric nano probe for Cd^2+^ detection (LOD = 27.8 nM), exhibited strong photocatalytic activity with 95% methylene blue degradation under sunlight, and showed enhanced antibacterial performance through synergistic interaction between the silver core and organic ligand. This work establishes a rational design strategy for multifunctional nanomaterials that integrate detection, degradation, and disinfection capabilities within a single platform.

## Abstract 2

A novel indole-imine Schiff base enabled one-pot synthesis of multifunctional AgNPs, serving as a Cd^2+^ colorimetric nanoprobe (LOD 27.8 nM), solar photocatalyst (95%), and enhanced antibacterial agent *via* silver-ligand synergy.

## Graphical abstract

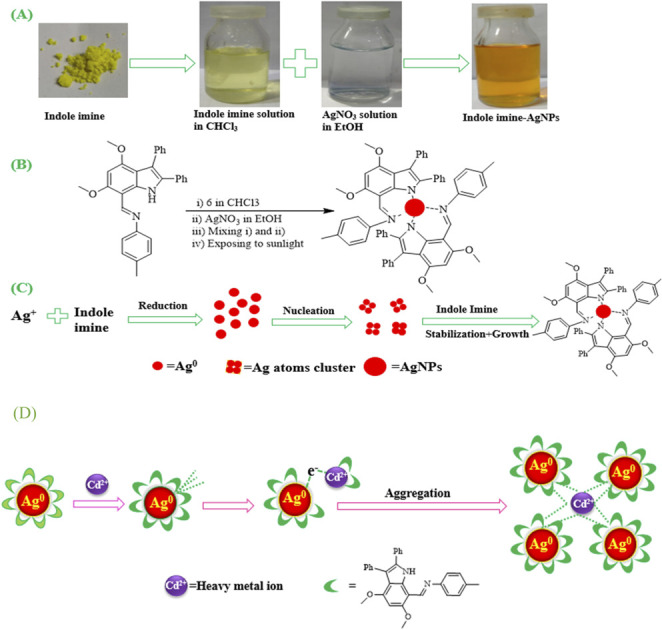

## 1. Introduction

The increasing challenges of water pollution and antimicrobial resistance (AMR) represent critical threats to global public health and ecosystem integrity.^1^ Several major factors contribute to these problems, including heavy metals,^2^ organic dyes,^3^ and pathogenic microbes.^4–6^ Among these, cadmium ions (Cd^2+^) are particularly concerning due to their extreme toxicity^7^ non-biodegradable nature,^8^ and tendency to bioaccumulate through the food chain, leading to renal damage, skeletal disorders, and carcinogenic effects in humans.^9–11^ Similarly, methylene blue (MB) persists in aquatic environments,^12–14^ where it inhibits photosynthesis and exhibits toxic effects on living organisms.^15–17^ Many antibiotics are no longer effective due to resistance mechanisms produced by pathogenic bacteria such *Escherichia coli*, *Staphylococcus aureus*, and *Pseudomonas aeruginosa*.^18–21^

In the fields of public health and environmental remediation, nanotechnology has become a potential candidate to overcome these issues.^22,23^ The NPs special physicochemical characteristics, including their high surface-area-to-volume ratio, adjustable optical properties, and increased reactivity,^24^ make them ideal candidates for tackling water pollution^25–28^ and antimicrobial resistance.^29^ Among different nanomaterials, metal-based NPs especially AgNPs have gained a lot of attention because of their efficacy and adaptability.^30,31^ With dimensions ranging from 1 to 100 nm, AgNPs have a significantly larger surface area-to-volume ratio likened to bulk Ag.^32,33^ Their nanoscale size conveys unique catalytic, electrical and optical properties.^34,35^ Strong plasmonic resonance and broad-spectrum activity enables AgNPs to interact with light and change color when they interact with particular pollutants due to aggregation.^36^ This property has been leveraged in the colorimetric detection of heavy metals such as Cd^2+^, Hg^2+^, and Pb^2+^ ions.^37–39^ Their semiconductor-like behavior under sunlight irradiation facilitates dye degradation through reactive oxygen species generation.^40–42^ The antibacterial qualities of AgNPs further increase their usefulness^43,44^

The capping agent critically dictates both the stability and sensing performance of nanomaterials.^45^ While plant-derived syntheses are common, their complex phytochemical mixtures hinder the identification of specific functionalities responsible for reduction, capping, and sensing, impacting reproducibility.^46^ In contrast, using defined synthetic capping agents allows for precise control over the nanoparticle's properties, enabling a clearer understanding of the formation mechanism and the rational design of selective sensors for heavy metal cations.^47^ For example, a study stated the synthesis of 4-(phenylsulphonamido)benzoic acid (PSBA)-functionalized AgNPs and demonstrated its selectivity to Ni^2+^.^48^ Similarly, another study reported the selectivity of hydroxyethylcellulose phthalate-capped AgNPs (HEC-PA@AgNPs) towards Hg^2+^.^49^ The AgNPs coated with chalcone carboxylic acid (CCA) serve as an optical indicator probe in the authors' selective and sensitive colorimetric approach for determining Cd^2+^.^50^ Thus, capping AgNPs with defined molecules provides precise control over their surface chemistry, dictating their sensing interactions with metal ions.

The indole (C_8_H_7_N) [2,3-benzo-(1*H*)-azole or 2,3-benzo-(1*H*)-pyrrole] is a highly common heterocyclic framework found in nature. It is a privileged structure in medicinal chemistry, renowned for its diverse pharmacological effects and role as a signaling molecule in biological systems.^51–53^ Several studies have utilized organic molecules for the synthesis and stabilization of AgNPs, but the use of a well-defined indole-imine Schiff base, particularly in a completely non-aqueous medium, remains rare.

This study introduces a strategically designed indole-imine Schiff base that serves a dual role as both a reducing and capping agent for the synthesis of silver nanoparticles (AgNPs) within a completely non-aqueous medium. This approach circumvents the inherent instability of aqueous synthesis, providing exceptional control over the nanoparticle surface and yielding a platform with superior colloidal stability. The precise molecular capping does not merely passivate the particles; it actively engineers their interface, granting them a unique trifunctionality.

## 2. Materials and methods

### 2.1. Materials

AgNO_3_, Ba(NO_3_)_2_, Ni(NO_3_)_2_·6H_2_O, Mn(NO_3_)_2_, Co(NO_3_)_2_·6H_2_O, Cd(NO_3_)_2_, Zn(NO_3_)_2_·6H_2_O, Al(NO_3_)_3_·9H_2_O, NaCl, NaOH, HNO_3_, methylene blue dye 7, CHCl_3_ and EtOH were purchased from Sigma-Aldrich, Fluka or Merck. The distilled water was obtained from Khushaab Water® plant at University of Sargodha. All the glass-ware was washed with dilute HNO_3,_ rinsed with distilled H_2_O and dried in an oven at 50 °C.

### 2.2. Synthesis of *N*-(4′-methylphenyl) (4,6-dimethoxy-2,3-diphenyl-1*H*-indol-7-yl)methanimine 6

#### 2.2.1. Synthesis of 2,3-diphenyl-(1*H*)-indole 3

The mixture of 4,6-dimethoxyaniline 1 (2.00 g, 13.1 mmol, 3 eq.) and benzoin 2 (2.78 g, 13.1 mmol, 3 eq.) was stirred constantly at 120 °C in reflux within a period of 2 h. The mixture was cooled to room temperature and the PhNH_2_ (0.41 g, 4.4 mmol, 1 eq.) and AcOH (8.5 g, 8.1 mL, 0.141 mol, 32 eq.) was added to the mixture. The blend turned to dark brown solution after 5 h of stirring at 130 °C. But on slowly cooling the solution to room temperature precipitation took place. Just a simple filtration of the precipitate gave a crude product of which a white amorphous solid (2.845 g, 66%) was the result when the crude product was washed with chilled MeOH. This amorphous solid was crystallized in a small portion using EtOAc. Spectroscopic and spectrometric characterization details of compound 3 are available in Section 1 of the SI.

#### 2.2.2. Synthesis of 4,6-dimethoxy-2,3-diphenyl-(1*H*)-indole-7-carbaldehydes 4

To the solution of POCl_3_ (1.38 g, 0.84 mL, 9 mmol, 3 eq.) in DMF (20 mL) at room temperature, the indole 3 (0.987 g, 3 mmol, 1 eq.) was added like drops with constant stirring. The resulting solution was then stirred at ambient temperature during an interval of 2½ h, after which a quench of chilled H_2_O (50 mL) and a basification of 50 mL of aq. NaOH solution of 1 M were added. This gave the result of a yellow precipitate that was filtered, rinsed with cold H_2_O and dried over anhydrous Na_2_SO_4_ and on a desiccated under reduced pressure to give the desired aldehyde (0.889 g, 83%) in the form of a yellow amorphous solid. A tiny bit of this amorphous solid was recrystallized EtOAc/MeOH and was used in spectroscopic and spectrometric identifications. Spectroscopic and spectrometric characterization details of compound 4 are available in Section 2 of the SI.

#### 2.2.3. Synthesis of indole imine 6

The mixture of 4,6-dimethoxy-2,3-diphenyl-(1*H*)-indole-7-carbaldehyde 4 (1.00 g, 2.8 mmol, 1 eq.) and 4-methylaniline 5 (390 mg, 3.64 mmol, 1.3 eq.) were dissolved in dry EtOH (25 mL) and refluxed till the aldehyde spot disappeared on neutralized (using elution with *n*-hexane/Et_3_N 1 : 1) TLC plate, which was spotted with 2,4-DNPH dip. The evaporation to dryness under reduced pressure was conducted to eliminate solvent, and solid product was purified by washing in cold MeOH (3 × 20 mL) and the residue obtained was a yellow amorphous solid 6 (1.06 g, 85%). Different solvents were used in recrystallization of this solid but no further purification resulted.

*R*_f_: 0.56 (CH_2_Cl_2_/*n*-hexane 3 : 2); MP: 180 °C; *ῡ* (cm^−1^) KBr: 3294 (N–H), 1581 (C=N); log *ε* (*λ*_max_ nm): 4.42422 (367); *δ*_H_ in ppm (400 MHz): 11.56 (bs, 1H, NH̲), 9.11 (s, 1H, H^8^), 7.20–7.42 (m, 14H, 3Ph), 6.23 (s, 1H, H^5^), 3.79, 3.97 (s, 3H each, OCH̲_3_), 2.38 (s, 3H, CH̲_3_); *δ*_C_ in ppm (100 MHz): 158.6, 159.2 (s, C^4^ & C^6^), 155.5 (d, C^8^), 150.3 (s, C^1^‴), 134.6 (s, C^4^‴), 136.6, 134.8 (s, C^7a^ & C^2^), 133.0, 132.5 (s, C^1^′ & C^1^″), 131.5, 129.7, 128.5, 127.8 (all 2×, d, C^3^′, C^3^″, C^2^′ & C^2^″), 127.4 (2×, d, C^2^‴, C^6^‴), 126.9, 126.0 (d, C^4^′ & C^4^″), 121.1 (2×, d, C^3^‴), 114.5, 113.2 (s, C^3^ & C^3a^), 102.2 (s, C^7^), 87.9 (d, C^5^), 55.4, 56.8 (q, 2OC̲H_3_), 20.9 (q, C̲H_3_).

### 2.3. Synthesis of Ag NPs of indole imine 6 (AgNPs-6)

A solution (2.5 mM) of indole imine 6 in CHCl_3_ was added like drops to a stirred solution of AgNO_3_ (50 mL of 2.5 mM in EtOH) in 5 minutes. The color of the solution turned to golden yellow from colorless during the procedure of stirring, which reflects to forming AgNPs. The solution was then covered with Al-foil and kept carefully.

To obtain pure AgNPs-6, the suspensions of the obtained AgNPs-6 was then centrifuged at 6000 rpm for 25–30 min in falcon tubes to pellet the AgNPs-6. The supernatant was decanted, and the pellet was re-dispersed in CHCl_3_. This washing procedure was repeated three times at 6000 rpm for 10 min to ensure complete removal of unreacted precursors. The final product was dried to a powder and then they were stored to be characterized and used in various activities.

### 2.4. Characterization

The UV-Vis and IR spectrum (as anhydrous KBr discs) were recorded by using UV-1800 (Shimadzu, Japan) and 8400S (Shimadzu, Japan) FT-IR, respectively, at the Central Research Laboratory, University of Sargodha, Sargodha (Pakistan). The ^1^H-NMR and ^13^C-NMR were operated on a Bruker AVANCE DPX (300 or 400 MHz) spectrometer in CDCl_3_ at the ICCBS, HEJ Research Institute of Chemistry, University of Karachi, Karachi (Pakistan) and for MS Q-TOF Ultima API (Micromass) facility in Biomedical Mass Spectrometry Facility (BMSF), UNSW, Sydney (Australia) was utilized. The SEM images were acquired using FEI Nova 450 Nano SEM (University of Peshawar, Pakistan) running at 30 kV. To analyze the crystalline structure of the synthesized NPs (NPs), the XRD technique was used. The single crystal XRD measurements were accompanied using Cu Kα radiation at a wavelength of 1.54056 Å, covering the 2*θ* range from 30° to 80°, which was recorded on Bruker Kappa APEX 11 CCD diffractometer at Department of Physics, University of Sargodha, Sargodha (Pakistan). The powder XRD analysis was performed using a JDX-3532 XRD instrument by JEOL, Japan. To execute DLS and ZP measurements at a fixed 173° scattering angle and 25 °C the Zeta-sizer Nano ZS (Malvern analytical) was used.

### 2.5. Effect of concentration of indole imine 6 and Ag^+^ ions on the synthesis of AgNPs-6

To optimize the reaction conditions, the experimental parameters which can affect AgNPs formation including the indole imine 6 dosage and Ag^+^ ions concentration were checked. UV-Vis spectroscopy was used to observe and establish the optimum conditions for the synthesis of AgNPs-6.

To observe the effect of concentration of indole imine 6 on the fabrication of AgNPs-6 was carried out by mixing Ag^+^ solution (10 mL, 2.5 mM) with 6 (10 mL, 1.5–2.5 mM) each and the UV-Vis spectra of all the obtained AgNPs-6 were recorded. In a similar manner, in order to determine the effect of different concentration of Ag^+^ ions on the synthesis of AgNPs-6, the reaction was performed by adding Ag^+^ ions solution with different concentration of (10 mL, 2.5–3.5 mM) to 6 (10 mL, 2.5 mM). The UV-Vis of all the synthesized AgNPs-6 were recorded.

### 2.6. Determination of optical bandgap energy (*E*_g_) of AgNPs-6 by Tauc plot

The optical bandgap energy (*E*_g_) of the AgNPs-6 was determined by using the Tauc plot (eqn (1)).

1 (*αhυ*)^*n*^ = *A*(*hυ* − *E*_g_)

here, *α*, *h*, *ν*, *A*, *E*_g_ and *n* represents the absorption co-efficient, Planck's constant, frequency, an energy independent constant, band-gap energy and nature of transition (*n* = 2) for direct band gap material, respectively. The factor “*hν*” denote energy of incident photon.^54^

Under the Tauc plot method we plot “*hυ*” over *x*-axis and “(*αhυ*)^2^” over *y*-axis where h is the Planck's constant and *υ* is the frequency of the incident radiation, A is the proportionality constant and *α* is the absorption coefficient as a function of wavelength *λ*. The *hυ* = 1240/*λ* of AgNPs-6, this value was taken on *x*-axis and the value of (*αhυ*)^2^ is calculated as (2.303 × absorbance × energy)^2^ and this value was taken on *y*-axis. On plotting “*hυ*” against “(*αhυ*)^2^”, a curve is obtained. The extrapolation of the line component of the curve is made by drawing a tangent line to the figure until it meets the *x*-axis. The optical band gap of the material is given by the point of contact of this tangent line and the *x*-axis.

### 2.7. Stability study of AgNPs-6

To check the effect of pH on stability of AgNPs-6, stock solution of water-soluble suspension of AgNPs-6 was prepared by suspending pure AgNPs-6 in distilled H_2_O. The pH was varied (2.0–8.0) and adjusted to desired target by using HNO_3_ (10% w/v) and NaOH (10% w/v). 5.0 mL solution of AgNPs-6 was mixed with 5.0 mL solution of pH (2.0–8.0) each and left standing for 24 h in the dark. After 24 hours the UV-Vis spectra of the sample were recorded. Each type of experimental sample was prepared in triplicate; therefore, the results represents the average of these samples.

Similarly, to check the degradation of AgNPs-6 with time, the UV-Vis spectra of synthesized AgNPs-6 were recorded direct after the synthesis and after 7, 15 and 30 days of synthesis. To check the effect of temperature on stability of AgNPs-6, the water-soluble suspensions of AgNPs-6 was heated to 100 °C on a hot plate and UV-Vis spectra was recorded after 30, 60 and 120 minutes. To check the impact of the electrolyte concentration on synthesized AgNPs-6 stability, various concentrations (0.01 M to 1.0 M) of NaCl solutions were added to the water soluble suspension of AgNPs-6 and the results were recorded through UV-Vis spectrometer.

### 2.8. Colorimetric detection of cations in aqueous solution by AgNPs-6

To test the AgNPs-6 capability to detect the metal ions, water-soluble suspension of AgNPs-6 (100 ppm) was made by dissolving 10 mg of pure AgNPs-6 in distilled water (100 mL). A 1.0 mM solution of multiple metal ions (Ba^2+^, Co^2+^, Zn^2+^, Cd^2+^, Al^3+^, Na^+^, Mn^2+^, and Ni^2+^) was added to an equal volume of water-soluble suspension of AgNPs-6. A color change in AgNPs-6 colloidal solution was noted and UV spectra of the mixtures were recorded for each.

For quantitative analysis, various concentrations from 0 to 210 nM of Cd^2+^ ions were added to AgNPs-6 suspension and the change in LSPR was observed by recording UV–Vis spectra of these colloidal suspensions. The limit of detection (LOD) of Cd^2+^ ions by AgNPs-6 was calculated by establishing a calibration curve between the absorbance and the concentration of Cd^2+^ ions and implying the eqn (2).2
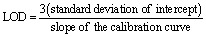


Moreover, a testing was run to determine whether there was any interference by the other metal ions when sensing Cd^2+^ ions. The study used the UV-Vis spectroscopic analysis of AgNPs-6 (1 mL) mixed with Cd^2+^ (1.0 mL of 500 µM) in the presence of Ba^2+^, Co^2+^, Zn^2+^, Al^3+^, Na^+^, Mn^2+^, and Ni^2+^ salts (1.0 mL of 1 mM).

To assess the response time of the nanosensor, a solution of Cd^2+^ at a concentration of 200 nM (1.0 mL) was mixed with 100 ppm of AgNPs-6 (1.0 mL). At various time intervals (0, 1, 2 and 6 min) the peak of LSPR was observed after mixing under the ideal circumstances.

Also, the ability of the proposed nano-probe to determine Cd^2+^ in river water as well as tap water samples was examined. The water sources used to collect water samples were the Jhelum River in the Sargodha district and tap water was collected from the Chemistry Laboratory of the University of Sargodha in Sargodha, Pakistan. Different amounts of Cd^2+^ ions were spiked into the water samples.

### 2.9. Photo-catalytic study of AgNPs-6

10 ppm solution of methylene blue 7 was prepared by dissolving 5.0 mg of methylene blue 7 in 500 mL of DW. The 25 mL of this 10 ppm methylene blue 7 dye solution was mixed with 20 mg of chemically synthesize AgNPs-6. Magnetic stirring was used to mix the given reaction suspension in darkness for 30 min to attain the equilibrium of the working solution before irradiation, whereas the UV-Vis spectra was taken prior to irradiation *i.e.* at 0 min. The successive scattering was then being exposed to daylight and UV-Vis spectra were taken after every 15 minutes (*i.e.* 15, 30, 45, 60, 75, 90 and 105 minutes) by taking little amounts of solutions. The percentage degradation of MB dye by AgNPs-6 was calculated using eqn (3).^55^3
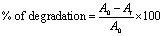
where *A*_0_ = absorbance prior to exposure and *A*_t_ = absorbance at *t* min.

The kinetic and thermodynamic studies of MB degradation by AgNPs-6 was also carried out at different temperatures (298, 308, 318, 333) K. Rate constant (*k*) and some of the thermodynamic parameters (*E*_a_, Δ*G*, Δ*S*, and Δ*H*) were also determined.^56^

Moreover, it was determined whether the AgNPs-6 could be again used as a photocatalyst for multiple times. The photo-catalyst AgNPs-6 was used to destroy the MB. After carrying out a of the first round of MB dye degradation, centrifugation of the sample was done in a falcon tube at a speed of 6000 rpm for 25 minutes to retrieve the photocatalyst. The liquid part was discarded. The AgNPs-6 were washed thrice using deionized water, and dried in an oven at 60 °C, 12 hours. Photocatalytic degradation of MB was again carried out through the use of dried up photocatalyst. The dye degradation and regeneration procedure was performed for five times. After every cycle, the degradation percent of dye was calculated.

Catalytic effectiveness of AgNPs-6 photocatalyst was further described by calculating the turnover frequency (TOF) and turnover number by eqn (4) and (5) given below.4
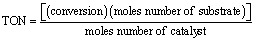
5
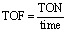
In order to determine how different species take part in deterioration, a study on radical scavenging was performed. Four scavengers were utilized in the experiment: *p*-benzoquinone (*p*-BQ) for ˙O_2_^−^, isopropanol (IPA) for ˙OH, (l)-ascorbic acid (l-AA) for H_2_O_2_, and disodium ethylenediamine acetate (Na_2_EDTA) for H^+^. For each test, 0.2 mM solution of the radical scavenger was mixed with a dye solution (30 mL of 10 ppm), along with a 20 mg catalyst. The degradation of each dye was measured using the method outlined above.

For every test 0.2 mM of the radical scavenger was mixed into a combination of a dye solution (30 mL of 10 ppm) and a 20 mg catalyst. The degradation of each dye was determined using the method outlined above.

### 2.10. Antimicrobial activity

A reported protocols were followed in introducing the disc diffusion approach in the determination of the antibacterial potential of the AgNPs-6 approach to four bacteria, [*Bacillus subtilis* (*B. subtilis*), *Escherichia coli* (*E. coli*), *Pseudomonas aeruginosa* (*P. aeruginosa*), and *Staphylococcus aureus* (*S. aureus*)].^57,58^ The AgNPs-6 suspension was made by adding 10 mg AgNPs-6 in 10 mL distilled water. The agar solution was prepared by dissolving 2.8 g of nutrient agar in 100 mL of distilled water. All the Petri plates, paper discs and solution of agar covered with Al-foil were autoclaved at 120 °C for 20 min. Once all Petri dishes were sterilized, 25 mL of the agar solution was added into the Petri dish and cooled at 70 °C. The Petri dishes were put in the room temperature till the agar solution hardened. Using the cotton buds the particular bacterial rinsings were then separately introduced into each Petri dish. After this, loaded discs were placed in Petri dishes, assigning an alphabetic letter to each of the disc. A 30 µL of DW (negative control), antimicrobial disc of Ceftriaxone (positive control), 30 µL of indole imine 6 solution and 30 µL of AgNPs-6 solution were loaded on each disc and were left in incubation at 37 °C for 24 h. The diameters of the zone of inhibition were determined in millimeters of both control and AgNPs-6 by the use of scale. The whole procedure was performed under a clean antiseptic laboratory atmosphere under the laminar flow cabinet.

## 3. Results and discussions

### 3.1. Synthesis of indole imine 6

**Scheme 1:**
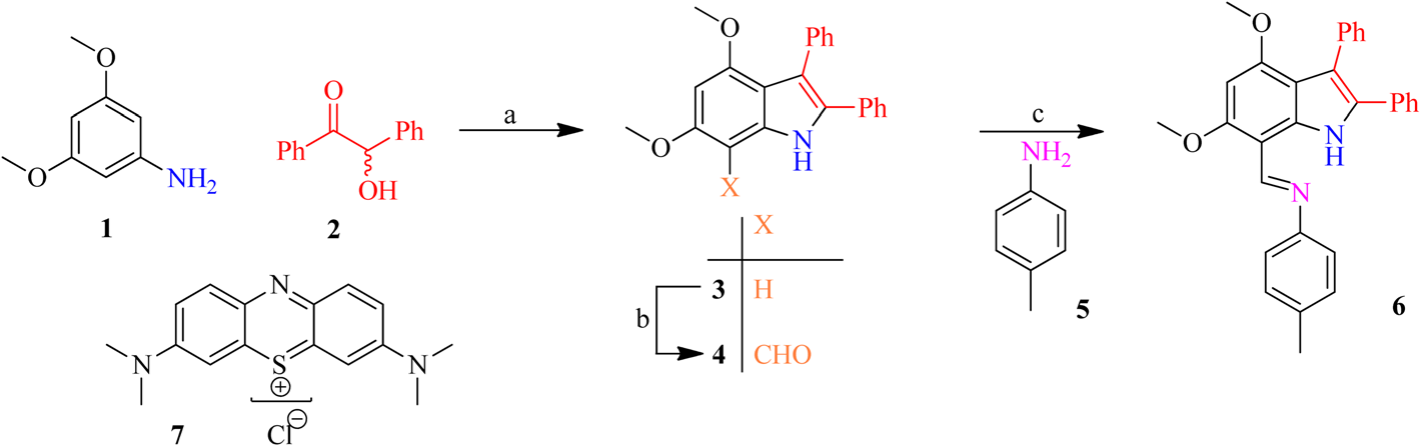
The synthesis of indole imine 6; (a) AcOH, PhNH_2_ (cat.), 130 °C (5 h); (b) POCl_3_, Me_2_NCHO, ambient; (c) EtOH, reflux.

An indole imine was synthesized following Bischer indole protocol. The commercially available 3,5-dimethoxyaniline 1 was reacted with benzoin 2 (±-2-hydroxy-1,2-diphenylethanone) to afford 2,3-diphenylindole 3. The resulting substituted indole 3 was subjected to formylation, through the Vilsmeier–Haack sequence, which involves the addition of POCl_3_ to DMF under anhydrous conditions to generate chloroiminium ion as an intermediate. This process subsequently furnished 2,3-diphenylindole-7-carbaldehyde 4. A coupling reaction was carried out between indole carbaldehyde 4 and 4-methylaniline 5 to furnish an indole imine (a Schiff base) 6 (Scheme 1).

The appearance of only one characteristic absorbance signal (N–H) at 3329 cm^−1^ and bathochromic shift in *λ*_max_ (308 to 324 nm) represents the creation of indole nucleus, which was further verified by comparing the recorded ^1^H and ^13^C NMR spectra with literature values. The Vilsmeier–Haack protocol was followed for the formylation of 2,3-diphenylindole 3 in which DMF was reacted with POCl_3_ to form chloroiminium ion intermediate that act as formylating agent. The orange-red colouration of the product on the TLC plate upon treatment with acidified 2,4-DNPH solution indicates the presence of aldehydic carbonyl C. A little change was observed in the N–H stretching between indole 3 (3329 cm^−1^) and 2,3-diphenylindole-7-carbaldehyde 4 (3296 cm^−1^) in FT-IR spectrum. The absence of an absorption band at ∼3100 cm^−1^ shows the absence of H-bonding between aldehydic O and indolic H. The aldehydic functional group was believed to be in extensive conjugation as C=O bending in carbaldehyde 4 emerges at low value (1608 cm^−1^). The introduction of formyl moiety to indolic chromophore increases the conjugation which was revealed by the bathochromic shift in the *λ*_max_ of 7-formylindole 4 (372 nm) is observed in contrast to indole 3 (324 nm). The formylation of indole 3 to &-formylindole 4 was also confirmed by loss of doublets of H^7^ in aromatic region (6.57 ppm) in ^1^H-NMR with the increase of other singlet at 10.41 ppm with no extending after D_2_O exchange. The ^13^C-NMR present C^5^ and C^7^ of indole 4 as doublet and quaternary carbons, respectively, which provides another proof of formylation at C^7^.

**Fig. 1:**
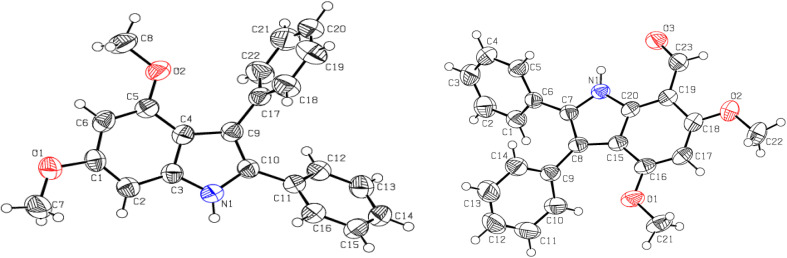
The ORTEP presentations of 4,6-dimethoxy-2,3-diphenyl-(1*H*)-indole 3 (left) and 4,6-dimethoxy-2,3-diphenyl-(1*H*)-indole-7-carbaldehyde 4 (right).

The formyl functionality having highly conjugated system was further ascertained by a doublet carbon of aldehydic functionality (H–C=O) appeared at 188.2 ppm in 7-formylindole 4. The formyl functionality shifted C^7^ to downfield as result of –I effect. According to single crystal XRD studies minimum stearic strain was maintained by perpendicular alignment of both phenyl groups in aldehyde 4 (Fig. 1 and Table S1).^59^ The EIMS of 7-formylindole 4 showed [M]_+_ as the base peak because it was more stable and no additional considerable fragmentation was achieved.

**Fig. 2:**
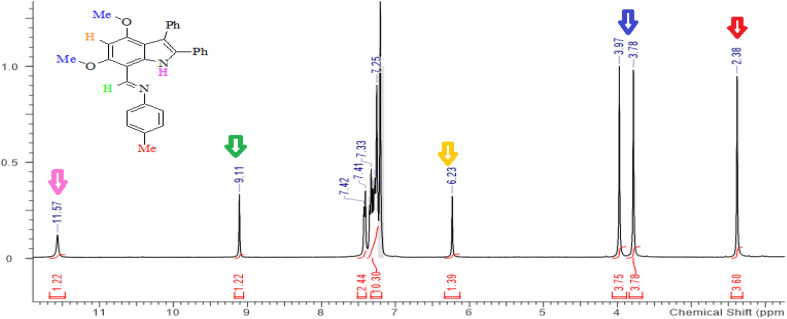
Complete ^1^H-NMR of indole imine 6 (300 MHz in CDCl_3_) with ^1^H integration, indicating the assignment of prominent ^1^Hs in different chemical environment.

The synthesized imine 6 exhibited no absorbance in the carbonyl range (1600–1850 cm^−1^), while a new peak was observed at 1581 cm^−1^ in FT-IR spectrum that corresponds to 〉C=NPh which was formed by the transformation of C=O. The signal at 3294 cm^−1^ indicates indolic N–H stretching. The imine 6 showed a light hypsochromic shift in the *λ*_max_ (367 nm) compared to their corresponding carbaldehyde 4 (372 nm), indicating a decrease in conjugation mainly due to steric factor of the 4-methylphenyl ring on iminic N. But the occurrence of a singlet of the most shielded aromatic proton (H^5^) in just imine 6 at 6.23 ppm, the appearance of an additional sharp singlet (do not show widening upon D_2_O exchange) at 9.11 ppm, the presence of a most deshielded new broad singlet (show widening upon D_2_O exchange) at 11.57 ppm in ^1^H-NMR (Fig. 2) and upfield shifting of an aldehydic methine C of indole-7-carbaldehyde 4 (188.2 ppm) to an iminic methine C (155.5 ppm) in ^13^C-NMR approves the formation of indole imine 6.

### 3.2. Synthesis of AgNPs-6

**Fig. 3:**
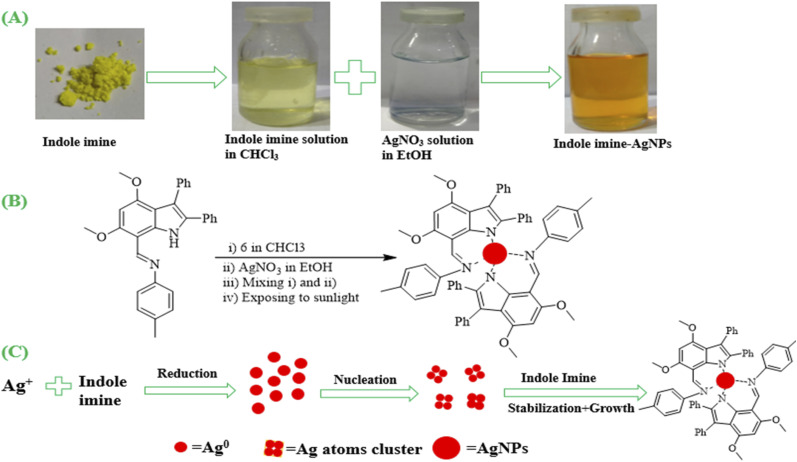
(A) Photographs of indole imine 6, solution of indole imine 6 in CHCl_3_, AgNO_3_ solution in EtOH and AgNPs-6, (B) synthesis scheme of indole imine 6 reduced AgNPs and (C) step-wise mechanism of synthesis of indole imine-AgNPs.

The indole imine 6, a synthetic organic compound, was utilized as reducing as well as capping agent to synthesize Ag NPs. A 2.5 mM solution of indole imine 6 prepared in CHCl_3_ was mixed drop-wise in 2.5 mM solution of AgNO_3_ prepared in EtOH with continuous magnetic stirring. The color of solution shifts from colorless to light yellow and then finally to golden yellow. The Fig. 3A shows photographs of powdered indole imine 6, solution of indole imine 6 in CHCl_3_, AgNO_3_ solution in EtOH and AgNPs-6. The characteristic color of reaction mixture changes from colorless to golden yellow, indicating that Ag^+^ ions are reduced to Ag^0^ and that AgNPs are formed. A color change show successful synthesis of AgNPs-6 owning to similar results reported in various researches.^60^

The formation of AgNPs-6 follows a sequence of steps, including reduction, nucleation, stabilization, and growth. Fig. 3B and C shows scheme for synthesis of indole imine 6 reduced AgNPs and step-wise mechanism for the synthesis of indole imine-AgNPs, respectively.

Step (i): comprises the formation of Ag^0^ atoms by reduction of Ag^+^ ions using indole imine 6.

6 AgNO_3_ + 6 → Ag^0^

Step (ii): succeeding the formation of Ag^0^ atoms, the nucleation progression starts, wherein Ag^0^ atoms aggregate to form tiny nuclei and afterward clusters as follows:

7 AgNO_3_ + 6 → Ag^0^(cluster)

Step (iii): after that, the functional group of indole imine 6 accelerates growth and stabilization by processes such as collision, fusion, adsorption of Ag^0^, and Ostwald ripening.

This eventually leads to formation of AgNPs-6. The adsorbed clusters contain the functional groups due to which the Ag^+^ ions were reduced to Ag^0^ atoms.

8 Ag^0^_m_ + (indole–C=N–ϕ) → (Ag^0^_m_)–(indole–C=N–ϕ)

where ϕ = phenyl ring.

The shapes and the size of the metal nanostructures are typically predetermined by the interaction between nucleation and growth processes that rely on the types of functional groups present in the precursor.

Examining the change in color of the solution and UV-Vis spectroscopy confirmed that AgNPs-6 had been formed. In this process, indole imine 6 acts as a capping agent, keeping AgNPs-6 stable in solution, and even after 15 days of preparation, no indication of adhering together was observed.

### 3.3. Characterization of indole imine 6 stabilized AgNPs-6

#### 3.3.1. UV-vis spectroscopy and bandgap energy (*E*_g_) by Tauc plot

**Fig. 4:**
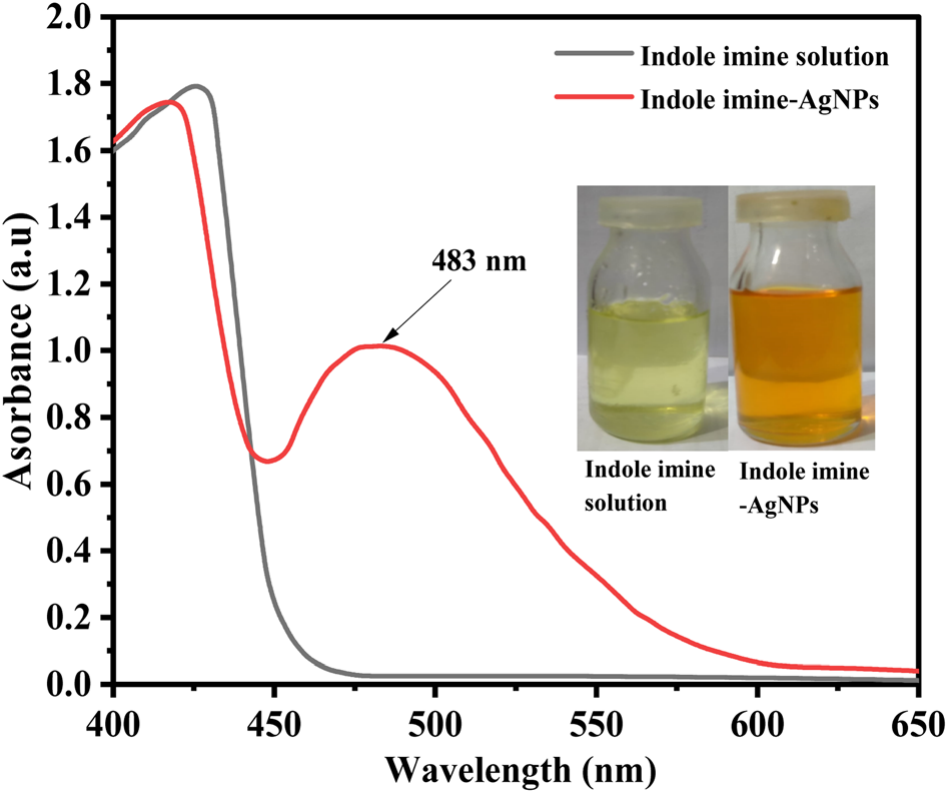
UV-Vis spectra and images (inset) of indole imine 6 solution and AgNPs-6.

The solution that has been prepared changing its color to light yellow during AgNPs-6 synthesis was a sign of successful synthesis, which was later verified by logging and comparing the UV-Vis spectrum of the indole imine 6 solution and the synthesized AgNPs-6 solution. The Fig. 4A presented the UV-Vis spectra of indole imine 6 and AgNPs-6. In the case of the indole imine 6 solution, no absorption band was seen, but the AgNPs-6 synthesis was evident due to the distinctive SPR absorption peak of AgNPs at 483 nm. Typical AgNPs produce SPR peaks with *λ*_max_ values between 390–490 nm.^61^

#### 3.3.2. FT-IR and XRD analyses

**Fig. 5:**
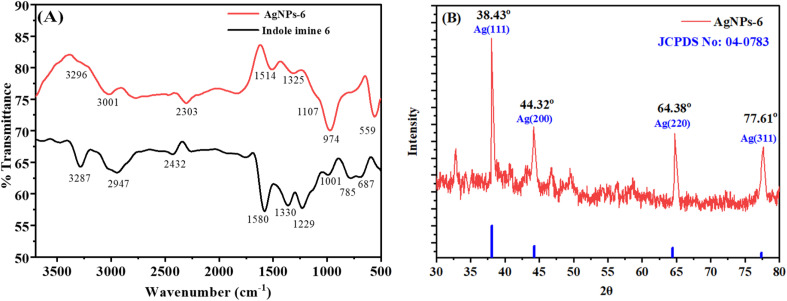
(A) Overlaid FT-IR spectra of indole imine 6 and AgNPs-6 and (B) PXRD diffraction pattern of AgNPs-6.

FT-IR analysis was used to determine functional groups present in indole imine 6 and on surface of AgNPs-6. The overlaid FT-IR spectra of indole imine 6 and AgNPs-6 are shown in the Fig. 5A. Table S2 shows a summary of the FT-IR peaks and the corresponding functional groups that they were assigned to. The indole imine 6 that is a synthetic indole derivative exhibited characteristics band having peak at 3287 cm^−1^ in FT-IR spectrum is referred to N–H stretching vibrations of indole. The peaks at 2947, 1330 and 1001 cm^−1^ corresponds to the Ar C–H, C=C and Ar C–C, respectively are stretching vibrations observed due to presence of aromatic groups in the indole imine 6. The signals at 1580 and 1229 cm^−1^ represents the C=N and C–O–C vibrations due to the presence of imune group and ether group, respectively. Peak observed at 687 cm^−1^ is characteristic peak due to deforming vibrations of C–H bonds. Nearly same characteristic bands of indole imine 6 are observed in another study previously.^62^

The spectral lines intensities of AgNPs-6 are quite different as compared to those of the indole imine 6. In the FT-IR spectrum of AgNPs-6 the shifting of strong N–H signal to a very weak signal at 3296 cm^−1^, C–H signal to 3001 cm^−1^, C=C signal to 1325 cm^−1^, the shifting of strong peaks of C=N and C–O–C to weak peaks at 1514 cm^−1^ and at 1107 cm^−1^ giving a strong evidences of donation of electrons by imine and ether groups to Ag, which reduced the imine and ether group's electron density and caused a lower wavenumber on the FT-IR spectrum (Table S2). In FT-IR spectrum of AgNPs-6, nearly all peaks were retained with only slight disturbance to wavenumber as well as emergence of another peak at 559 cm^−1^ due to presence of weak M-NR interactions in the spectrum of these NPs. This justify the strong involvement of the imine (RC=NR) group of indole imine 6 in binding with sp orbital of Ag atoms of AgNPs *via* a lone pair of N atoms for their reduction and covering and hence bringing in the successful fabrication and stabilization of AgNPs-6.^48^

Thus, in summary, the AgNO_3_ lattice was separated by coordination with indole imine 6 and subsequently dissolved in CHCl_3_.

PXRD of AgNPs-6 is shown in Fig. 5B. This PXRD spectrum results also supports that AgNPs-6 are formed. The XRD was observed with strong reflections at 2*θ* of 38.03°, 44.32°, 64.38° and 77.61° for (111), (200), (220) and (311) crystal facets respectively. The face-centered cubic (fcc) coordination of AgNPs-6 reflects these reflections (JCPDS data file no. 04-0783). These experimental values are consistent with the past literature concerning capped AgNPs.^63,64^ In addition, the mean size of the crystallites of AgNPs was obtained by Debye Scherrer equation as presented in eqn (9).^65^9
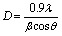
where *D* is the crystallite size, *λ* is the full width at half maximum, *θ* is the diffraction angle, and (Cu κα = 0.154 nm) is the source wavelength. The average crystallite size, we found was 19.2 nm. The discovery that the crystallite particle size is smaller than the total particle size suggests that the little crystallite particles were formed. Other crystal parameters which are the dislocation density, micro strain, and degree of crystallinity were also estimated using the dislocation density (*δ*) eqn (10), the micro strain (*ε*) formula eqn (11), and the degree of crystallinity relation eqn (12). The calculated crystal parameters are given in Table S3.10
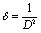
11
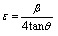
12
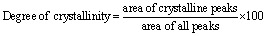


#### 3.3.3. SEM and EDX analysis

**Fig. 6:**
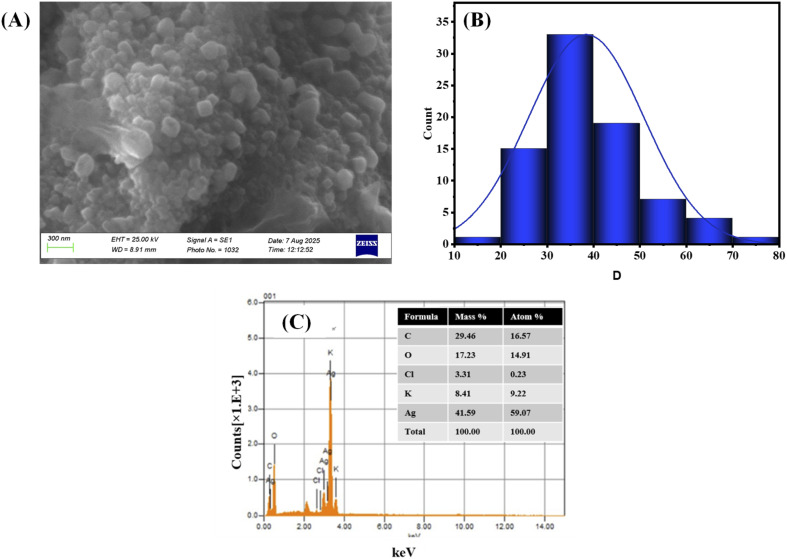
(A) Micrograph of SEM of AgNPs-6 (B) particle size distribution histogram of AgNPs-6 and (B) an EDX image (inset shows elemental composition of AgNPs-6).

Morphology of synthesized AgNPs-6 was inspected with the help of scanning electron microscopy (SEM). In the SEM image provided in Fig. 6A, the well separated AgNPs-6 with very minute visible aggregations can be visualized. The ImageJ software calculated the size of NPs *i.e.*, 38.1 ± 4.03 nm (Fig. 6B). In order to study the synthesis of indole imine 6 stabilized Ag NPs, the Energy-Dispersive X-ray Spectroscopy (EDX) was performed and the spectra obtained justified the synthesis of the particles as intense characteristic peaks at 3 keV. These peaks are the confirmation of synthesis and stabilization of indole imine 6 stabilized AgNPs as illustrated in Fig. 6B. There were also a few more peaks of C, O, Cl, and K. A C and an O peak were observed because one of the precursor molecules, indole imine 6 is mostly carbon and oxygen. Peaks of Na/K can be noted where detector and grid applied in EDX make use of structures containing these elements. The spectra also indicate the presence of the Cl peak which may have occurred due to the addition of contaminants in NPs during production, handling or in the analysis process or due to the existence of impurities in any of the precursors or water used in preparing solutions. The elemental composition of AgNPs-6 is shown in inset of Fig. 6B.

#### 3.3.4. DLS and zeta potential measurements

**Fig. 7:**
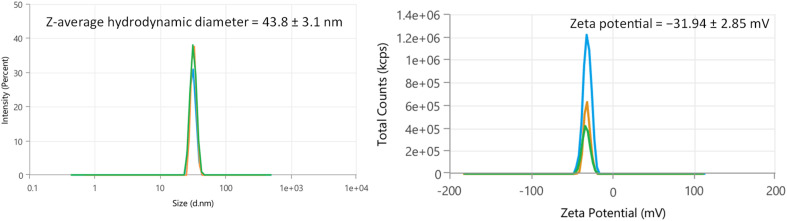
(A) DLS measurements and (B) zeta potential of AgNPs-6.

The size distribution of the synthesized AgNPs-6 was estimated using dynamic light scattering (DLS) that falls between 20 and 80 nm. The synthesized mean hydrodynamic particle size of AgNPs-6 is about 43.8 ± 3.1 nm (Fig. 7A). The different colored curves in Fig. 7 represent triplicate measurements of the same AgNPs-6 sample, confirming the reproducibility of the hydrodynamic size distribution.

The apparent discrepancy between the average crystallite size from XRD (19.2 nm) and the hydrodynamic diameter from DLS (43.8 ± 3.1 nm) is a characteristic feature of capped NPs. The Scherrer equation applied to the XRD data provides the size of the individual crystalline domains of metallic silver. In contrast, the DLS measurement reports the overall hydrodynamic diameter, which includes the inorganic Ag core, the organic capping layer of indole-imine 6, and the associated solvation shell that diffuses with the particle in solution. The larger DLS size thus provides direct evidence of the successful functionalization and colloidal nature of the AgNPs-6.

Nature and magnitude of surface charge of NPs are credited with the stability and aggregation of NPs. The zeta potential measurements, consisting of the surface charges of fabricated NPs is an important parameter to obtain a clue about stability and mechanism of coagulation of NPs. Surface charge magnitudes of NPs actually associates with the degree of repelling forces between NPs and therefore inhibits the aggregation. A zeta potential between 0 to ±5 mV indicates a high rate of NPs aggregation, the range ±10 to ±30 mV represent low stability therefore being prone to form aggregates and while ±30 to ±40 mV indicates fairly good and average stability of the NPs. NPs are grades as perfectly stable based on their zeta potential measurements within a range of ±40 to ±60 mV or higher.^66^ The zeta potential measurements were taken for AgNPs synthesized and stabilized by indole imine 6, a synthetic organic compound. As prepared NPs exhibited zeta potential values −31.9 ± 2.8 mV for AgNPs-6 as shown in Fig. 7B. The large negative charges on the NP surface can explain the repulsive forces between these NPs that do not allow them to aggregate together, as prepared NPs and they will be in proper dispersion in a solution. Therefore zeta potential values provide very powerful concern on effective production of well stable AgNPs-6.

### 3.4. Effect of concentration of 6 and Ag^+^ ions on the synthesis of AgNPs-6

The dosage of indole imine 6 and concentration of Ag^+^ ion on the formation of AgNPs-6 was studied, with a gradual redshift of *λ*_max_ with the concentration, which supports the change in the particle sizes and the population. The best conditions were found to be 2.5 mM of indole imine 6 and AgNO_3_. Extensive spectral and discussion is given in the SI (Fig. S1A and B).

### 3.5. Stability of AgNPs-6 under various conditions

AgNPs-6 was characterized by excellent stability at different pH, durability, high temperatures and moderate ionic strength. Aggregation was only notable to occur under acidic conditions (≤4) and high concentrations of NaCl (1.0 M), and insignificant spectral variations were found after 15 days, 100 °C, and 0.1 M NaCl. In-depth UV-Vis data and discussion are given at the SI (Fig. S2A–D). Table S4 shows relative change in *λ*_max_ and absorption intensity of AgNPs-6 under different conditions.

### 3.6. Selective colorimetric sensing of metal ions using AgNPs-6

The sensing ability of the AgNPs-6 was explored with respect to examining its interaction with Ba^2+^, Co^2+^, Zn^2+^, Cd^2+^, Al^3+^, Na^+^, Mn^2+^, and Ni^2+^ ions. After mixing a corresponding amount of AgNPs-6 water-soluble suspension with each metal ion solution (1 mM), the UV-Vis spectra were collected.

**Fig. 8:**
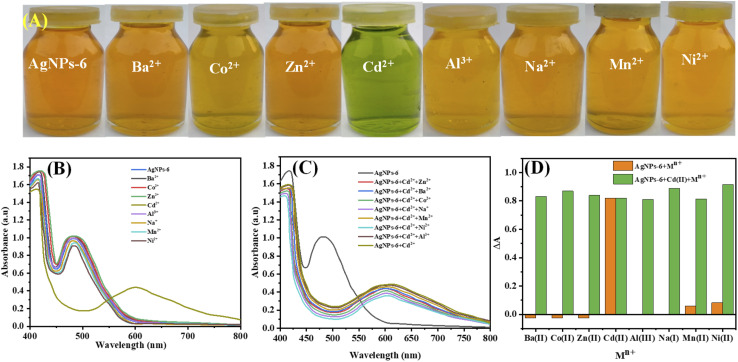
(A) Photographs of AgNPs-6 solutions containing different metal ions, (B) UV-Vis spectra of AgNPs-6 when mixed with various metal ions and (C and D) UV-vis spectra and a bar graph of corresponding change in absorbance of AgNPs-6 with different metal ions with respect to the control solution, respectively.

With the exemption of Cd^2+^, where the mixtures' color changed from golden yellow to green (Fig. 8A), the typical LSPR peak at 483 nm of AgNPs on the UV-Vis was also reduced and a new peak of 603 nm was developed as shown in Fig. 8B. The change in the color of mixture of other metal ions and Ag NPs and the change in UV-Vis spectra of the mixture were not observed as in Fig. 8A and B. This implies that these 6-functionalized AgNPs show high discrimination to the detection of Cd^2+^. The principles of selective sensation of the present metal ions in solutions had already been founded by the change of the color and LSPR peak observed in the case of Cd^2+^.

#### 3.6.1. Interference study

Additionally, a study was carried out to observe the potential interference caused by different metal ions while Cd^2+^ ions are being sensed. The procedure includes the measurement of UV-Vis spectra of AgNPs-6 (1 mL) mixed with Cd^2+^ (500 µM, 1 mL) in the presence of Ba^2+^, Co^2+^, Zn^2+^, Al^3+^, Na^+^, Mn^2+^, and Ni^2+^ (1 mM, 1 mL). As shown in Fig. 8C, it is confirmed that even in the existence of higher concentrations of various metal ions solutions, there is a comparable decrease in the absorbance of the spectra. This alteration was limited to Cd^2+^ ions. This is an indication that AgNPs-6 had selectivity in the interaction with Cd^2+^ ions, and no interaction existed between AgNPs-6 and the other metal ions in the salt solution. The findings of the change in AgNPs-6 absorbance (Δ*A*) are shown in Fig. 8D. The green bars represent the change in absorbance of AgNPs-6 + 1 mM solution of each Ba^2+^, Co^2+^, Zn^2+^, Cd^2+^, Al^3+^, Na^+^, Mn^2+^, and Ni^2+^. While the orange bars show the absorbance change of AgNPs-6 + Cd^2+^ in the presence of 1 mM solution of Ba^2+^, Zn^2+^, Co^2+^ Al^3+^, Na^+^, Mn^2+^, and Ni^2+^. One can note that the absorbance of UV-Vis spectra changes almost equally at even higher concentrations of other salt solutions. This indicates that the AgNPs-6 have sensitivity and selectivity in the detection of Cd^2+^.

To confirm that the colorimetric response originates from the NPs rather than the organic ligand alone, a critical control experiment was performed. When indole-imine 6 alone was exposed to Cd^2+^ ions under identical conditions, no spectral shift was observed in Fig. S4 (SI). This definitive result confirms that the sensing mechanism is specifically mediated by the AgNPs-6 platform, where Cd^2+^ induces nanoparticle aggregation through interactions with the surface-bound ligand, rather than through solution-phase reactions with free ligand molecules.

#### 3.6.2. Selective detection of Cd^2+^ ions using colorimetric assay

**Fig. 9:**
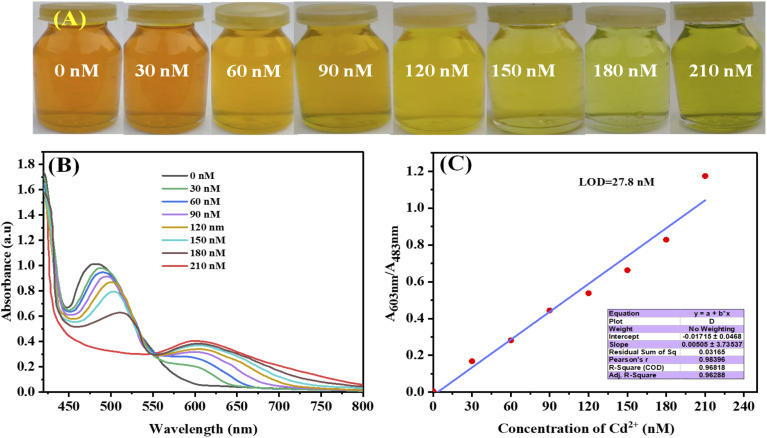
(A) Photographs depicting color change with various concentrations of Cd^2+^, (B) UV-Vis graphs of AgNPs-6 in the presence of different concentrations of Cd^2+^ and (C) calibration curve drawn between the absorbance *A*_603nm_/*A*_483nm_ and the concentration of Cd^2+^.

Various concentrations of Cd^2+^ (0–210 nM) were added to the solution of AgNPs-6 for quantitative examination. Interestingly, this technique allowed for the identification of Cd^2+^ ions with the unaided eye. As the amount of Cd^2+^ rises, the images in Fig. 9A depict the visual color extinction of AgNPs-6. This color change was measured by taking and analyzing the UV-Vis spectra of these solutions. As the concentration of Cd^2+^ ions grew, the UV-Vis absorption at 603 nm slowly increased while the UV-Vis absorption at 483 nm gradually dropped, as shown in Fig. 9B.

A calibration curve was created between the intensity ratio *A*_603nm_/*A*_483nm_ and the concentration of Cd^2+^ a respectable degree of linearity was attained within the range of 0–210 nM, with a correlation value (*R*^2^) of 0.9682 (Fig. 9C). Eqn (2) was used to calculate the LOD, which came out to be 27.8 nM which was much lesser than maximum contaminant level of Cd^2+^*i.e.* 45 nM (5 µg L^−1^) in drinking water by United State Environmental Protection Agency (EPA).^67^

#### 3.6.3. The suggested mechanism of the interaction between of heavy metal ions and AgNPs-6

To study how Cd^2+^ is detected by AgNPs-6, various concentrations of Cd^2+^ were added to the water-soluble AgNPs-6. The resulting colloidal suspensions' UV-visible spectra were then used to evaluate the shift in LSPR. The absorption at 483 nm gradually decreased as the Cd^2+^ ions increased, accompanied by a red shift and the appearance of a new peak at 603 nm. This conduct implies nanoparticle aggregation induced by an increase in Cd^2+^ concentration. We hypothesize that the red shift in *λ*_max_ resulted from the Cd^2+^-mediated aggregation of AgNPs-6, which served as the foundation for this detecting nano-probe.

The selectivity of AgNPs-6 for Cd^2+^ over other tested metal ions (Ba^2+^, Co^2+^, Zn^2+^, Al^3+^, Na^+^, Mn^2+^, and Ni^2+^) is attributed to the specific coordination chemistry between the Cd^2+^ ion and the organic functional groups of the indole imine 6 capping agent. The indole imine 6 presents specific binding sites, most notably the imine nitrogen (–C=N–) and the methoxy oxygen atoms (–OCH_3_), which are known to act as Lewis basic donors. The Cd^2+^ ion, with its soft Lewis acid character and a strong tendency to form stable complexes with N- and O-donor ligands, can effectively bridge adjacent AgNPs-6 by coordinating to these functional groups. This cross-linking leads to the aggregation of nanoparticles.

While ionic radius plays a role in the steric feasibility of complex formation, the superior selectivity for Cd^2+^ is primarily due to its optimal binding affinity and coordination geometry with the specific donor atoms presented by the capping layer. Other metal ions either form weaker complexes (*e.g.*, Zn^2+^, Mn^2+^) or have different preferred coordination environments, preventing efficient cross-linking and thus resulting in a negligible colorimetric response.

**Fig. 10:**
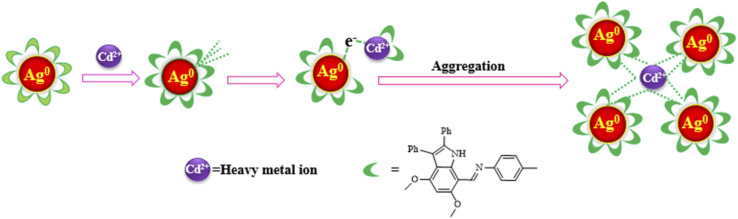
Proposed mechanism (Cd^2+^ mediated aggregation of AgNPs-6) for the detection of Cd^2+^ ions using AgNPs-6.

Therefore, we conclude that a Cd^2+^-specific coordination-driven aggregation is the most probable mechanism for the colorimetric sensing of Cd^2+^ ions utilizing AgNPs-6. Fig. 10 depicts the recommended mechanism for the detection of Cd^2+^ ions using AgNPs-6.

In order to compare the sensing efficiency of the developed colorimetric AgNPs-6 with others reported so far a comparison in terms of colorimetric detection of Cd^2+^ using similar types of AgNPs is tabulated in Table 1 below. The performance of AgNPs-6 as a colorimetric sensor for Cd^2+^, with its low LOD and high selectivity, is competitive with many reported probes (Table 1).

**Table 1:** Comparison of AgNPs stabilized by different methods as a colorimetric sensing probe for Cd^2+^ determination

Surface stabilizing media	Linear range (µM)	LOD (µM)	Reference
Sulfoanthranilic acid dithiocarbamate functionalized AgNPs	10–100	5.8	68
Grape juice	0–150	4.95	69
Allium sativum stabilized Ag NPs (AgNPs-AS)	0–150	0.277	70
Chalcone carboxylic acid (CCA)	0.227–3.18	0.13	50
1-Amino-2-naphthol-4-sulfonic acid functionalized Ag NPs (ANS-AgNPs)	1.0–10	0.087	71
l-cysteine functionalized Au–Ag NPs	0.4–38.6	0.044	72
Secnidazole functionalized Ag NPs (SEC-AgNPs)	5.0–27	0.021	73
1,13-bis(8-quinolyl)-1,4,7,10,13-pentaoxatridecane modified AgNPs	0.5–6.0	0.016	74
5-Sulfosalicylic acid functionalized AgNPs	0.05–1.1	0.003	75
Indole imine-6 stabilized AgNPs	0–0.21	0.0278	This study

#### 3.6.4. Response time effect on colorimetric assay

During the 6 minute time period, after the mixing of 200 nM Cd^2+^ (1 mL) and 100 ppm AgNPs-6 (1 mL), progressive decrease in absorbance was observed and an increase in the *λ*_max_ at which the maximum LSPR band reached, shifting from 483 nm to 603 nm as described in Fig. S3 (SI). Throughout this period, the solution underwent a color transformation from yellow to green due to AgNPs-6 aggregation. After a duration of 6 min, the shape and intensity of the LSPR peak reached a state of stability and did not undergo any major changes. The rapid response time of AgNPs-6 in detecting Cd^2+^ makes it a good option for real-time research.

#### 3.6.5. Real sample analysis

The Table S5 contains the results of water samples recovery and the relative standard deviations (RSDs) of the actual water samples. The spiked water revealed higher than 85% recovery rates, corresponding to an RSD around 4%. These results imply that the colorimetric nano-probe has a good probability of qualifying as a dependable and accurate method of Cd^2+^ detection in actual water samples.

### 3.7. Photocatalytic degradation of methylene blue 7

The cationic dye methylene blue 7 was degraded with the help of the AgNPs-6 by photocatalysis.

**Fig. 11:**
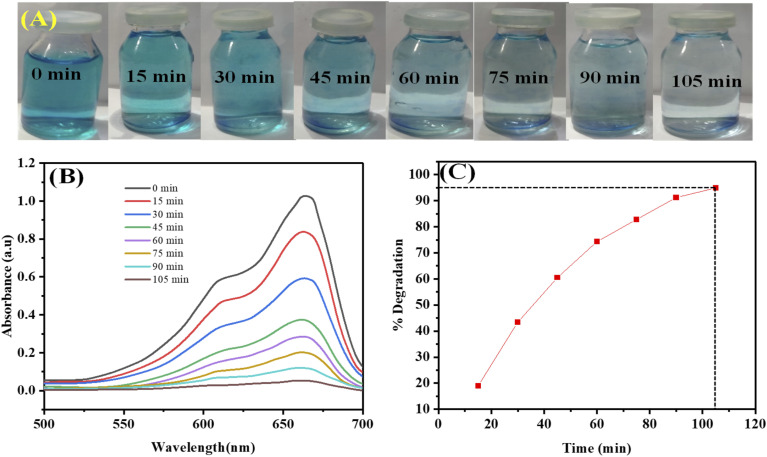
(A) Photographs of visual illustration of change of color, (B) plot of UV-Vis spectra of photocatalytic degradation and (C) plot for % degradation of methylene blue 7 dye using AgNPs-6 at different time intervals.

The dye's dark blue color changes to a lighter blue and finally turns almost colorless after being visible to daylight for 105 minutes, as seen in Fig. 11A. The color changed from dark blue to lighter blue and then almost colorless, indicating degradation. During the revelation of the AgNPs-6 added dye 7 solution, UV Vis spectra were obtained at regular intervals (0, 15, 30, 45, 60, 75, 90, and 105 min). The Fig. 11B depicts the steady fall and final diminishment of the characteristic dye 7 absorption peak, which is positioned at 664 nm, signifying the complete photocatalytic destruction of dye 7.

The essential photocatalytic role of the AgNPs was verified through a control experiment using only the indole-imine 6 ligand under identical illumination conditions. The Fig S5 (SI) shows that ligand alone demonstrated negligible photocatalytic activity, establishing that the observed 95% degradation efficiency is exclusively mediated by the AgNPs-6 composite. This result confirms that the AgNPs serve as the primary photocatalytic engine, while the organic capping layer likely contributes by modifying the electronic properties and enhancing visible light absorption.

#### 3.7.1. Kinetic study of dye 7 degradation

**Fig. 12:**
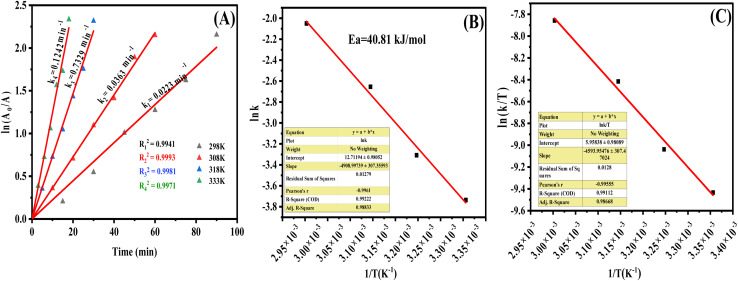
(A) Plot of first order kinetics of photocatalytic dye 7 degradation at different temperatures, (B) plot of ln *k versus* 1/*T* and (C) plot of ln *k*/*T versus* 1/*T* for the degradation of dye 7 by AgNPs-6.

The degradation of dye 7 followed pseudo-first-order kinetics at all studied temperatures, with rate constants increasing from 298 to 333 K. Thermodynamic analysis showed a moderate activation energy (40.81 kJ mol^−1^), an endothermic process (positive Δ*H*), decreased disorder (negative Δ*S*), and a non-spontaneous nature (positive Δ*G*). Complete kinetic plots, Arrhenius/Eyring analyses, and calculated parameters are provided in the SI (Fig. 12A–C and Table S6).

#### 3.7.2. Regeneration of the photocatalyst

Results of dye 7 degradation by performing five consecutive cycles of regeneration of AgNPs-6 photocatalyst are shown in Fig. S6. The slight reduction of these results by 10% is the hint that AgNPs-6 could be recycled effectively and successfully in respect of risky dyes degrading and wastes water treatment, and it does not suffer considerable loss of its catalytic activity. Furthermore, catalytic efficiency can also be described by active sites, turnover frequency (TOF), and turnover number (TON). The collection of locations where catalytic reactions take place can be described by the quantity of active sites. TON, which can be described as the catalyst's performance in the catalytic process, is just a number without a unit. TON of AgNPs-6 for dye 7 degradation was found to be 0.16 and 0.82 at 15 minutes and 105 minutes, respectively.

Conversion is one of them; it describes how much a catalyst degrades substrate molecules. TOF, a metric used to investigate reaction rates, is frequently used to describe the quantity of reaction cycles that take place over time.

Similarly at 15 and 105 minutes, the TOF of AgNPs-6 for dye 7 degradation was determined to be 0.011 min^−1^ and 0.008 min^−1^, respectively.

#### 3.7.3. Proposed mechanism of photocatalytic dye degradation

The visible-light-driven activity of the AgNPs-6 is attributed to plasmon-mediated processes at the NPs surface. Upon light irradiation, the localized surface plasmon resonance (LSPR) of AgNPs induces collective oscillations of conduction electrons, which decay to generate energetic “hot” charge carriers. These hot electrons possess sufficient energy to participate in surface redox reactions before thermal relaxation. Additionally, the coordination interaction between indole imine and surface silver atoms creates an interfacial environment that facilitates efficient charge transfer between the plasmon-excited Ag surface and adsorbed molecular species. This interaction enhances the lifetime and utilization of plasmon-generated charge carriers by promoting electron transfer to molecular oxygen, leading to the formation of reactive oxygen species (ROS) such as superoxide radicals. Simultaneously, the remaining positive charge on the Ag surface contributes to oxidative processes at the interface. The synergistic effect of plasmon-induced hot electron generation and ligand-assisted interfacial charge transfer is responsible for photocatalytic efficiency.^75–77^

### 3.8. Antibacterial activity

**Fig. 13:**
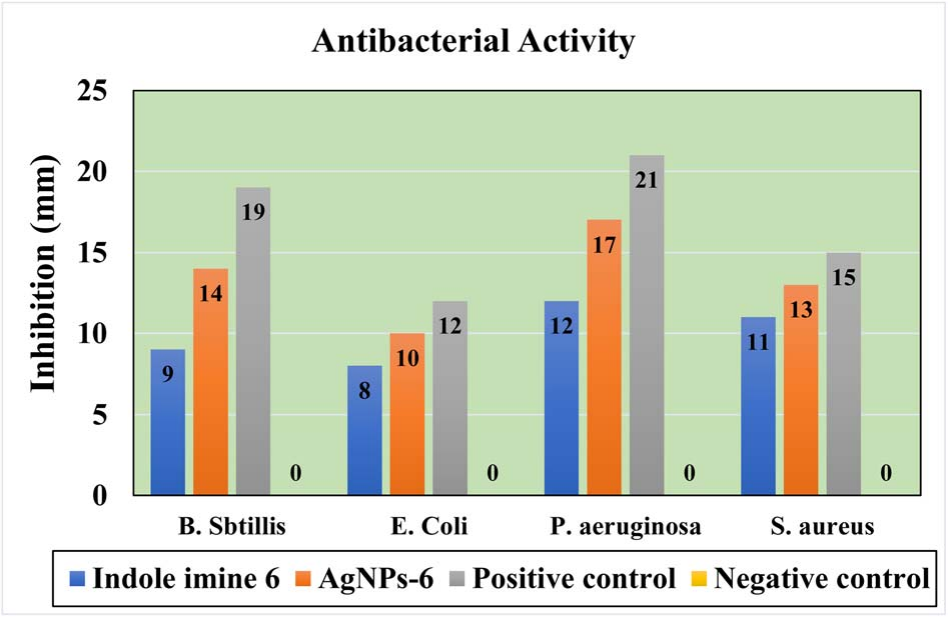
Graphical illustration of antibacterial activity of indole imine 6, AgNPs-6 positive control and negative control against different bacterial strains.

Metallic NPs' mechanism of action indicates how strongly they bind to bacterial cell membranes. The strong reducing characteristics of the metallic NPs cause the main functional groups of liposaccharides and cell membrane proteins to break down. Alternatively, they are oxidized by oxygen found inside cells, which causes oxidative damage through the Fenton process. The pathogenic organisms die as a result of structural damage caused by the translocation of NPs across the lipid bilayer of the cell.^78^ The antibacterial activity of indole imine 6 stabilized AgNPs was investigated utilizing the disc diffusion method against four different species of harmful bacteria *i.e. Bacillus subtilis*, *Escherichia coli*, *Pseudomonas aeruginosa* and *Staphylococcus aureus*. The produced AgNPs-6 has antibacterial potentials on both Gram-positive and Gram-negative bacteria. The antibacterial activity of AgNPs-6 is notably enhanced compared to the indole-imine 6 alone, indicating a synergistic effect between the Ag core and the organic ligand. Fig. 13 display the millimeter-scale diameter of the zones of inhibition surrounding each disc that contains NPs solutions and the control (positive and negative).

## 4. Conclusion

In summary, we have successfully developed a trifunctional nanomaterial through a rational design strategy. The use of a defined indole-imine Schiff base in a non-aqueous medium yielded AgNPs-6 with exceptional stability. This functionalization was quite effective in enhancing the colloidal stability of these AgNPs-6 even in pH range of 2.0 to 8.0, in higher ionic strength electrolytes (NaCl) as well as higher temperatures (100 °C) based on the zeta potential and UV-Vis values. Beyond demonstrating competitive performance in any single application, the primary innovation of this work is the integration of high-efficacy colorimetric sensing, visible-light photocatalysis, and synergistic antibacterial activity into a single, simply synthesized platform. Applications of these AgNPs-6 in a variety of mechanisms were examined; colorimetric nanoprobe, photocatalyst of an azo dye 7 (methylene blue) degradation and antibacterial agent. It served as a highly sensitive colorimetric probe for Cd^2+^ with a low detection limit of 27.8 nM and practicality in real water samples, a reusable solar photocatalyst that degraded 95% of methylene blue within 105 minutes primarily *via* superoxide radicals and holes, and a potent antibacterial agent whose activity was enhanced by a synergistic effect with the indole-imine capping ligand. This multifunctionality underscores the material's potential for integrated environmental remediation applications. This multifunctionality positions AgNPs-6 as a promising, economical candidate for developing integrated water treatment strategies where detection, pollutant degradation, and microbial disinfection are required simultaneously.

## Author contributions

Muhammad Kashif: methodology, investigation, formal analysis, writing – original draft. Abdul Rauf Razaa: conceptualization, supervision, project administration, validation, writing – review & editing, formal analysis. Shoaib Akhtar: writing – review & editing, formal analysis. Khaled Fahmi Fawy: funding acquisition, writing – review & editing. Muhammad Sher: writing – review & editing, formal analysis. Umar Nishan: writing – review & editing, formal analysis. Muhammad Imran Irfan: writing – review & editing, formal analysis. Azhar Abbas: conceptualization, supervision, project administration, validation, writing – review & editing, formal analysis.

## Conflicts of interest

The authors declare that they have no known competing financial interests or personal relationships that could have appeared to influence the work reported in this paper.

## Supplementary Material

RA-016-D5RA07704D-s001

## Data Availability

The data supporting this article have been included as part of the supplementary information (SI). Supplementary information: spectroscopic and spectrometric characterization data for compound 3. Spectroscopic and spectrometric characterization data for compound 4. Table S1: the crystallographic data of indole 3 and indole-7-carbaldehyde 4. Table S2: the FT-IR spectral analysis of indole imine 6 and AgNPs-6. Table S3: crystal parameters of AgNPs-6. S1.1: effect of concentration of 6 and Ag⁺ ions on the synthesis of AgNPs-6. S1.1.1: effect of dosage of 6 on AgNPs-6 synthesis. S1.1.2: effect of Ag⁺ ions concentration on AgNPs-6 synthesis. S1.2: stability of AgNPs-6 under various conditions. Fig. S1: (A) UV-Vis spectra showing the effect of indole imine 6 concentration and (B) Ag⁺ ions concentration on the synthesis of AgNPs-6. Fig. S2: (A) effect of pH, (B) effect of time period of 15 days, (C) effect of 100 °C temperature for 120 minutes and (D) effect of NaCl (0.01 M–1 M) on UV-Vis spectra of AgNPs-6. Table S4: Relative change in *λ*_max_ and absorption intensity of AgNPs-6 under different conditions. Fig. S3: response time effect on colorimetric assay in terms of change in absorbance of AgNPs-6 in the presence of 200 nM Cd^2+^. Fig. S4: UV-Vis spectra of Indole imine 6 when mixed with Cd^2+^ ions. Table S5: results for detection of Cd^2+^ ions using AgNPs-6 in real water samples. Fig. S5: UV-Vis spectra of photocatalytic degradation of methylene blue 7 dye using Indole imine 6 at different time intervals. S1.2.1: kinetic study of dye 7 degradation. S1.2.2: thermodynamic studies. Table S6: rate constant (*k*) and thermodynamic values of degradation of dye 7 by AgNPs-6. S1.2.3: suggested mechanism of photocatalytic dye degradation. Fig. S6: regeneration results showing the effectiveness of the AgNPs-6 photocatalyst for dye 7 break down after five repeated operations and regeneration cycles. DLS Report (Dynamic Light Scattering data). See DOI: https://doi.org/10.1039/d5ra07704d.
