# Supplementary material for: Synthesis of trifunctional indole-imine-based Ag NPs as a molecular probe for selective colorimetric detection of Cd(ii), photo-catalytic and antimicrobial agent
Source: RSC Adv. 2026 Feb 18;16(11):9631–47. doi: 10.1039/d5ra07704d (PMC12915638; doi:10.1039/d5ra07704d)
Supplement: RA-016-D5RA07704D-s001 [file RA-016-D5RA07704D-s001.pdf]

Supplementary Information (SI) for RSC Advances.
This journal is © The Royal Society of Chemistry 2026

Synthesis of Trifunctional Indole-Imine-Based Ag NPs as a Molecular Probe for Selective Colorimetric Detection of Cd(II), Photo-catalytic and Antimicrobial Agent

Muhammad Kashif [1], Abdul Rauf Raza [1], Shoaib Akhtar[1], Khaled Fahmy Fawy[2], Muhammad Sher [1], Umar Nishan [3], Muhammad Imran Irfan [1], Azhar Abbas [1,4,*]*

[1]Ibn e Sina Block, Institute of Chemistry, University of Sargodha, Sargodha-40100, Pakistan

[2] Department of Chemistry, Faculty of Science, Research Center for Advanced Materials Science (RCAMS), King Khalid University, P.O. Box 960, Abha, 61421, Saudi Arabia.

[3]Department of Chemistry, Kohat University of Science and Technology, Kohat 26000 KP Pakistan

[4]Department of Chemistry, Government Ambala Muslim College, Sargodha-40100, Pakistan

**Corresponding authors: rauf.raza@uos.edu.pk, azhar.ramzan@uos.edu.pk

Prof Dr Abdul Rauf Raza- Ibn e Sina Block, Institute of Chemistry, University of Sargodha, Sargodha-40100, Pakistan; https://orcid.org/0000-0002-4053-4862

Azhar Abbas- Ibn e Sina Block, Institute of Chemistry, University of Sargodha, Sargodha 40100, Pakistan; Department of Chemistry, Govt Ambala Muslim College, Sargodha-40100, Pakistan; https://orcid.org/0000-0002-5741-7136

1. Spectroscopic and spectrometric characterization data for compound **3**

R_f: 0.65 (EtOAc/*n*-hexane 1:1); MP: 240-242 °C; ῡ (cm^{-1}) KBr: 3329 (N-H); log ε (λ_{max}. nm): 4.1875 (324); t_R (min): 22.48; EIMS *m/z* in amu (rel. abundance %): 329 [$M^{+\bullet}$, 100], 314 ($M^{+\bullet}$ - $Me^{\bullet}$, 54); δ_H in ppm (300 MHz): 8.16 (bs, 1H, N<u>H</u>), 7.28-7.47 (m, 10H, 2 Ph), 6.78 (d, *J* = 1.9 Hz, 1H, H^7), 6.28 (d, *J* = 1.9 Hz, 1H, H^5), 3.87, 3.73 (s, 3H each, 2 $OC\underline{H_3}$); δ_C in ppm (75 MHz): 157.8, 155.3 (s, C^4 & C^6), 137.4, 135.9 (s, C^{7a} & C^2), , 133.0, 131.9 (s, $C^{1'}$ & $C^{1''}$), 131.5, 128.5, 127.8, 127.3 (all 2×, d, $C^{3'}$, $C^{3''}$, $C^{2'}$ & $C^{2''}$), 126.9, 125.9, (d, $C^{4'}$ & $C^{4''}$), 115.0, 113.0 (s, C^3 & C^{3a}), 92.5, 86.5 (d, C^7& C^5), 59.8, 56.4 (q, 2 $O\underline{C}H_3$).

2. Spectroscopic and spectrometric characterization data for compound **4**

R_f: 0.55 (*n*-hexane/ EtOAc 1:1); MP: 181 °C; ῡ (cm^{-1}) KBr: 3296 (N-H), 1635 (C=O); log ε (λ_{max}. nm): 4.1963 (372); t_R (min): 26.39; EIMS *m/z* in amu (rel. abundance %): 357 ($M^{+\bullet}$, 100); δ_H in ppm (300 MHz): 10.59 (bs, 1H, D_2O exchangeable, N<u>H</u>), 10.41 (s, 1H, D_2O non-exchangeable, H^8), 7.23-7.36 (m, 10H, 2 Ph), 6.15 (1H, s, H^5), 3.81, 4.00 (s, 3H each, 2 $OC\underline{H_3}$); δ_C in ppm (75 MHz): 188.2 (s, C^8), 163.0, 161.7 (s, C^4 & C^6), 136.8, 135.4 (s, C^{7a} & C^2), 133.3, 132.2, (s, $C^{1'}$ & $C^{1''}$), 131.3, 128.5, 127.9, 127.5 (all 2×, d, $C^{3'}$, $C^{3''}$, $C^{2'}$ & $C^{2''}$), 127.3, 126.2 (d, $C^{4'}$ & $C^{4''}$), 114.7, 112.7 (s, C^3 & C^{3a}), 104.2 (q, C^7), 86.9 (d, C^5), 56.4, 55.5 (q, 2 $O\underline{C}H_3$).

Table S1. The crystallographic data of indole **3** and indole-7-carbaldehyde **4**.

Parameters	*Crystalographic data*	
	*Indole **3***	*Indole-7-carbaldehyde **4***
Molecular Formula	$C_{22} H_{19} NO_2$	$C_{23}H_{19}NO_3$
Molecular mass [amu]	329.38	357.39 amu

Crystal System	Monoclinic	Monoclinic
a, b, c [Å]	11.7435(16), 9.4480(12), 15.940(2)	32.036 (2), 7.2010 (4), 17.3062 (8)
α, β, γ [º]	90, 106.682(7), 90	90 112.060 (2) 90
Volume of crystal [Å^3]	1694.2(4)	3700.1 (4)
Z	4	8
Density of crystal (calc.) [g/cm^3]	1.291	1.283
μ (MoKa) [/mM]	0.083	0.085
F (000)	696	880

Table S2. The FT-IR spectral analysis of indole imine **6** and AgNPs-**6**

*Indole imine **6***		*AgNPs-**6***	
ύ (cm^{-1})	*Functional group*	*ύ (cm^{-1})*	*Functional group*
3287	N-H	3296	N-H
2947	C-H str	3001	C-H
1580	C=N	1514	C=N
1330	C=C	1325	C=C
1229	C-O-C	1107	C-O-C
1001	C-C	974	C-C
687	C-H def.	559	Ag---N

Table S3. Crystal parameters of AgNPs-**6**

Sample	*Average crystalline size 'D' (nm)*	*Dislocation density $\delta \times 10^{-3}$ (nm^{-2})*	*Micro strain $\varepsilon \times 10^{-3}$*	*Degree of Crystallinity (%)*
AgNPs-**6**	19.2	2.7	0.67	96.8

S1.1. Effect of concentration of 6 and Ag^+ ions on the synthesis of AgNPs-*6*

To optimize the reaction conditions, the experimental parameters which can affect AgNPs formation including the **6** dosage and Ag^+ ions concentration were checked.

*S1.1.1. Effect of dosage of **6** on AgNPs-**6** synthesis*

As the indole imine **6** dosage was increased from 1.5 to 2.5 mM, a characteristic local surface plasmon resonance (LSPR) peak for AgNPs-**6** appeared for each sample. The λ_{max} was shifted towards a higher wavelength with a hyperchromic shift in absorbance as the indole imine **6** dosage was increased from 1.5 to 2.5 mM. The values of λ_{max} were at 471, 477, 480, 482 and 483 nm for 1.5, 1.75, 2.0, 2.25 and 2.5 mM for indole imine **6** doses, respectively. The reason behind this red shift in λ_{max} lies in the fact that for 1.5 mM of indole imine **6** was mixed with a certain constant concentration of Ag^+ ions (2.5 mM), there were less number of functional groups in the indole imine **6**. So, less number of smaller sized AgNPs-**6** were formed making LSPR peak shorter in λ_{max} with less absorbance. While as the doze of indole imine **6** for the same concentration of Ag^+ ions was increased, large number of functional groups are available thereby forming higher number of larger sized Ag NPs shifting λ_{max} to a higher wavelength with higher absorbance. The effect of indole imine **6** dosage on UV-Vis spectra is presented in Fig. **S1A**.

S1.1.2. Effect of Ag^+ ions concentration on AgNPs-6 synthesis

As the Ag^+ ions concentration was enhanced from 2.5 to 3.5 mM, then a characteristic local surface plasmon resonance (LSPR) peak for AgNPs appeared for each sample. The λ_{max} was shifted towards a higher wavelength with a hypochromic shift in absorbance as the concentration of Ag^+ ions was increased from 2.5 to 3.5 mM. The values of λ_{max} were at 483, 487, 488, 490 and 492 nm for 2.5, 2.75, 3.0, 3.25 and 3.5 mM Ag^+ ions solution, respectively. The reason behind this red shift in λ_{max} lies in the fact that for 2.5 mM Ag^+ ions solution mixed with a certain dose of indole imine **6** (2.5 mM), there were certain number of ions to form certain number of smaller sized AgNPs-**6** making LSPR peak shorter in λ_{max}. While as the concentration of Ag^+ ions for the same dosage of indole imine **6** was increased, smaller AgNPs-**6** aggregate to from larger number of AgNPs-**6** shifting λ_{max} to a higher wavelength with less absorbance. The influence of the concentration of Ag^+ ions on UV-Vis spectra of AgNPs-**6** is presented in Fig. **S1B** (SI).

As the maximum absorbance were witnessed while using 2.5 mM of each indole imine **6** and $AgNO_3$ as shown in Fig. S1A & S1B, so these are the optimum concentrations for the synthesis of AgNPs-**6**.

S1.2. Stability of AgNPs-6 under various conditions

Charge on the surface of NPs changes with pH change of the dispersion medium. This change in charge decides the stability of NPs in a specific dispersion medium [1]. For evaluation of the stability of the AgNPs-**6** in dispersion media of different pHs, the AgNPs-**6** were dispersed in media of different pHs ranging from 2 to 8 and their UV-Vis spectra were noted (Fig. **S2A**) (SI). It is obvious from the UV-Vis spectra that at acidic pH, there is less absorbance, and an increase in absorbance was witnessed in moving from pH values 5 to neutral to alkaline. Maximum absorbance was seen at pH=8. This can be attributed to the fact that at acidic pH i.e., at pH $\leq$ 4, the proton in the dispersion media neutralized the negative charges on the surface of the AgNPs-**6** (as evident from -32.9±1.3 mV zeta potential of AgNPs-**6** and the Ag NPs got aggregated thereby decreasing absorption intensity, which in turn decreases the stability of AgNPs-**6**. While as the pH of the dispersion media increased from 5 onward, there is no protonation of the functional

groups on the surface of the AgNPs-**6** and negatively charged NPs repel each other making the suspension stable. At alkaline pH, relatively larger NPs are disintegrated into smaller ones causing repulsion among the NPs thereby increasing the concentration of the NPs of smaller size. This increases the absorption intensity which in result increase the stability of the AgNPs-**6**. The relative change in absorption intensity is presented in Table **S2** (SI).

To determine the stability of AgNPs-6, Uv-Vis spectra of AgNPs-6 were measured after 5, 10 and 15 days of its formation as depicted in Fig. **S2B** (SI). The negligible difference in peak intensity of the 6-capped AgNPs even after 15 days also indicated the effectiveness of the use of indole imine **6** as capping and stabilizing agent.

The stability of AgNPs-**6** has also been determined through boiling the water-soluble suspension of nanoparticle at higher temperatures of 100 °C for 2 hours during which their UV-Vis spectra were recorded directly as indicated in the Fig. **S2C** (SI). AgNPs-**6** showed the insignificant change of absorbance intensity, which indicated the high stability of NPs even at high temperatures.

The electrolyte serves to counteract a charge on the surface of the NPs and when it is too large, this leads to aggregation of the NPs. Stability of NPs was determined as a change in absorption intensity of the NPs. Very minute decrease in the UV-Vis spectra (Fig. **S2D**) (SI) with an increase in the concentration of NaCl to 0.1 M can be regarded as the evidence of the exemplary stability of the NPs in such solution. The stability of NPs is however reduced significantly with increase in the concentrations of NaCl to 1.0 M as shown by the significant reduction in the LSPR intensity.

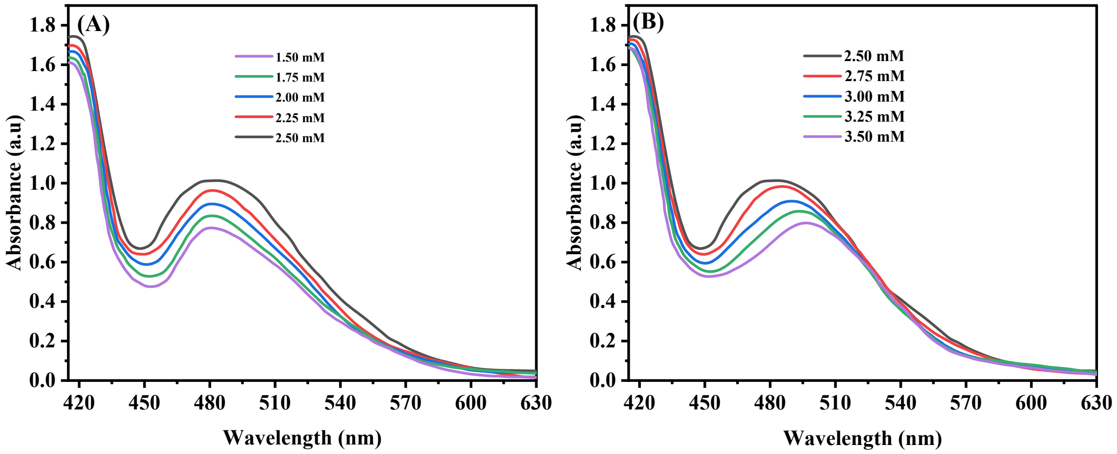

Fig. S1: (A) UV-Vis spectra showing the effect of indole imine **6** concentration and (B) Ag^+ ions concentration on the synthesis of AgNPs-**6**.

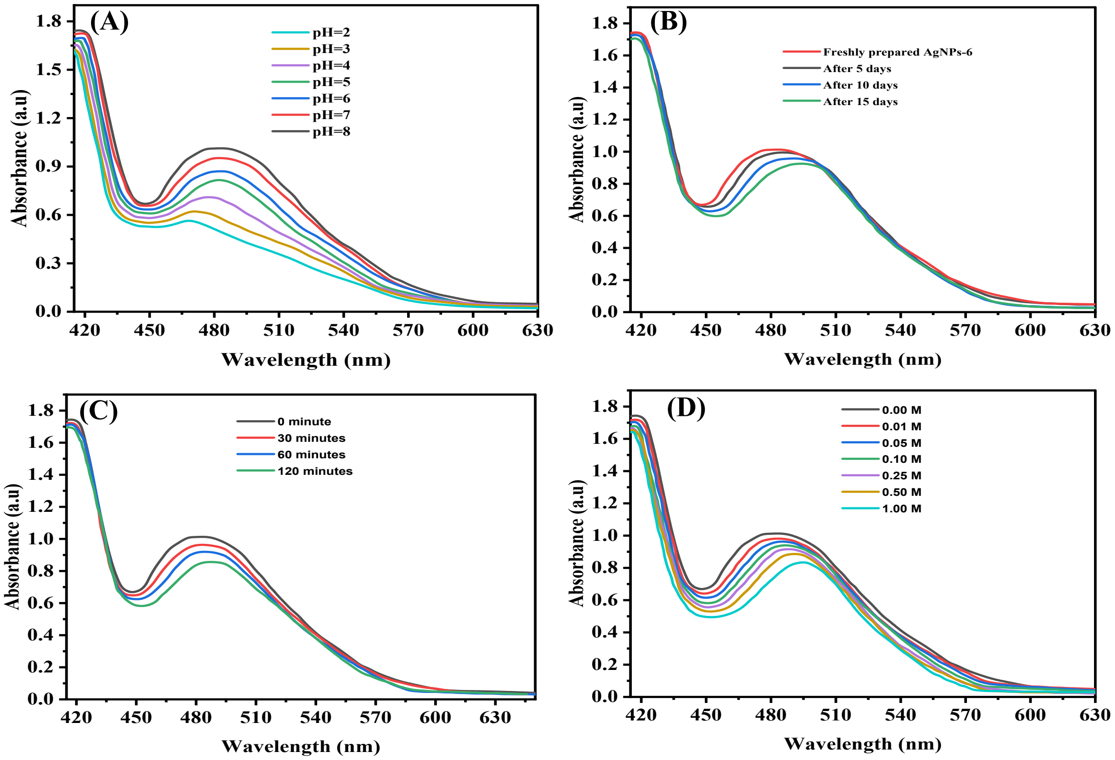

Fig. **S2**: (A) Effect of pH, (B) effect of time period of 15 days, (C) effect of 100 °C temperature for 120 minutes and (D) effect of NaCl (0.01M-1M) on UV-Vis spectra of AgNPs-**6**.

Table S4. Relative change in λ_{max} and absorption intensity of AgNPs-**6** under different conditions.

Variable	*Condition*	λ_{max} *(nm)*	*Relative Absorbance change (%)*
pH	2	469.1	44.1
	3	471.0	38.4
	4	475.3	29.8
	5	477.1	18.9
	6	477.9	12.4
	7	479.3	5.8
	8	482.4	0.2
Days	5	479.8	2.6
	10	486.9	5.7
	15	489.3	8.8
Minutes in 100 °C temperature	30	486.7	5.3
	60	488.7	9.8
	120	492.0	15.8
NaCl (M)	0.01	485.0	3.2
	0.05	485.5	4.9
	0.10	485.9	6.9
	0.25	486.6	9.6
	0.50	488.1	12.8
	1.00	492.6	17.8

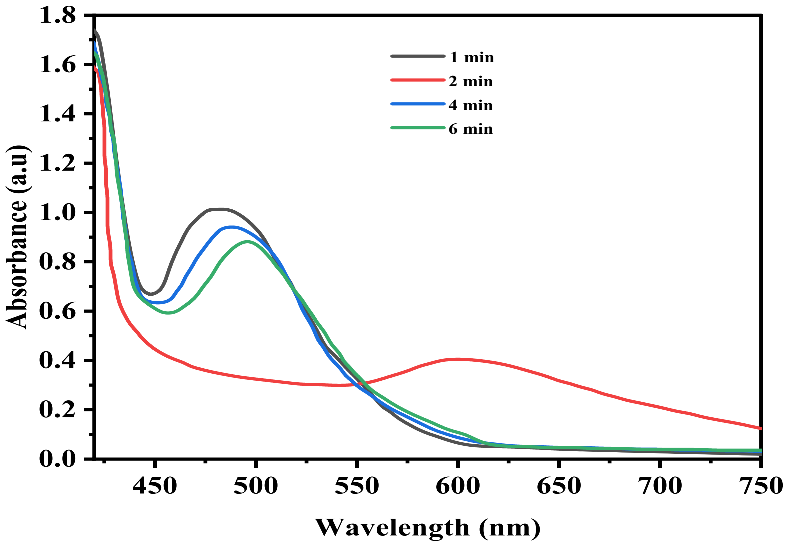

Fig**. S3**: Response time effect on colorimetric assay in terms of change in absorbance of AgNPs-6 in the presence of 200 nM Cd^{2+}.

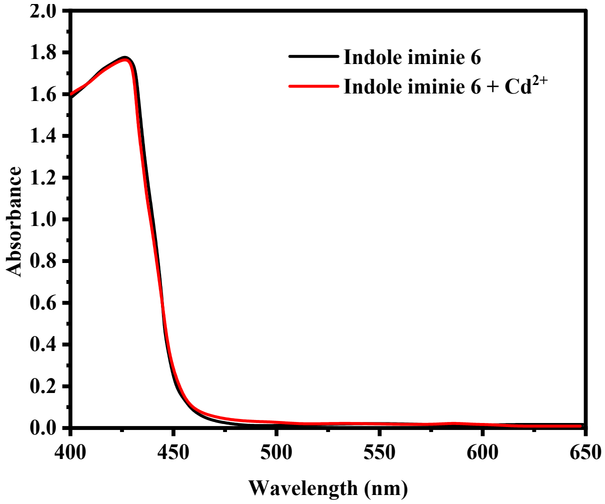

Fig. **S4**: UV-Vis spectra of Indole imine 6 when mixed with Cd^{2+} ions.

Table S5. Results for detection of Cd^{2+} iona using AgNPs-**6** in real water samples

	River Water		Tap Water	
Cd^{2+} added (nM)	Cd^{2+} found (nM)	Recovery (%)	Cd^{2+} found (nM)	Recovery (%)
30	24.18	80.60	24.46	81.53
60	48.93	81.55	49.3	82.17
90	74.62	82.91	75.13	83.48
120	101.03	84.20	101.95	84.96
150	128.24	85.49	129.31	86.21
180	156.82	87.13	158.36	87.98
210	188.49	89.76	189.65	90.31
Average recovery (%)	84.52		Average recovery (%)	85.23
Standard deviation	3.22		Standard deviation	3.18
RSD %	3.81		RSD %	3.73

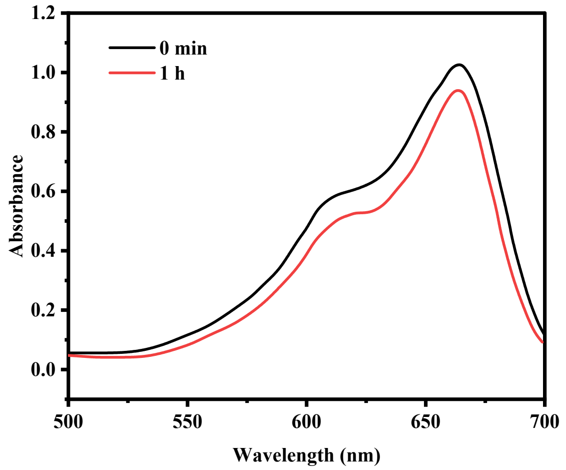

Fig. **S5**: UV-Vis spectra of photocatalytic degradation of methylene blue **7** dye using Indole imine **6** at different time intervals.

S1.2.1. Kinetic study of dye 7 degradation

Kinetics of reaction were studied using equation (S1) given below.

$$\ln\left(\frac{A_0}{A_t}\right) = kt \qquad (S1)$$

The k is the apparent rate constant of the degradation reaction, A_0 and A_t are the absorbance of the dye **7** at 0 and t time respectively. The Fig. **12A** shows the graphs of $\ln(A_0/A_t)$ versus reaction time for dye **7** break down at different temperatures. The slopes of the linear fitted lines were used to calculate the rate constants (k). The k values for the dye **7** degradation reaction at different temperatures are shown in Table 6. It is observed that the data is more or less linearly fit in a decent manner implying that degradation of dye 7 occurs according to pseudo-first-order. Additionally, at 298, 308, 318 and 333 K, the linear fits with R^2 approaching unity are 0.9941, 0.9993, 0.9981, and 0.9971 respectively, which is supporting even further the idea that dye **7** deterioration occurs according to pseudo-first-order kinetics.

S1.2.2. Thermodynamic studies

Difference of rate constant (k) with temperature at (298, 308, 318 and 333) K was used to calculate the activation energy (Ea) and other thermodynamic parameters. The activation energy of the degradation was calculated using the Arrhenius equation (S2).

$$lnk = -\frac{Ea}{RT} + lnA \qquad (S2)$$

Where k is the rate constant of reaction, A is the Arrhenius factor and R is the gas constant (8.314 J mol^{-1} K^{-1}).

The activation energy could be determined by plotting the graph of lnk versus 1/T (Fig. 12B) and determine activation energy as the slope of the straight line. The activation energy was medium at 40.81 kJ/mol. This figure indicated that the oxidation reaction was taking place with low activation barrier. The outcomes show that the photocatalytic breakdown of MB is a physical process. The value of ΔS and ΔH of activation were found out by using Eyring-Polanyi equation (S3).

$$\ln\left(\frac{k}{T}\right) = -\frac{\Delta H}{RT} + \frac{lnk_B}{h} + \frac{\Delta S}{R} \qquad (S3)$$

Where

k = Rate constant (min^{-1}) k_B = Boltzmann constant (1.3806×10^{-23} m^2 kg $s^{-2}K^{-1}$)

ΔH = Enthalpy (kJ / mol) R = Universal gas constant (8.314 J / mol K).

T = Temperature (K) H = Plank's constant 6.626×10^{-34} m^2 kg/s

and ΔS = Entropy (kJ / mol K)

The graph of lnk/T vs 1/T in Fig. **12C** gave a straight line, and the values of ΔS and ΔH can be obtained from the intercept and slope, respectively, and the value of ΔG can be calculated from the equation (S4):

$$\Delta G = \Delta H - T\Delta S \qquad (S4)$$

Where

ΔG = Free energy (kJ / mol) ΔH=Enthalpy (kJ / mol)

T = Temperature (K) and ΔS = Entropy (J / mol K)

Table S6 contains the values of rate constant k, Ea and thermodynamic parameters (ΔH, ΔS, and ΔG) calculated by the utilization of above mentioned equations. The large positive value of ΔH indicates the endothermic relationship of dye **7** degradation reaction. The ΔS of degradation is negative and has an implication that loss of disorder occurred in the action of degrading dye **7** and that a source of energy would be necessary to perform the reaction.

The value of the ΔG is positive and greater than 82.3 kJ mol^{-1}, which indicates that the reaction is not spontaneous.

Table S6. Rate constant (k) and thermodynamic values of degradation of dye **7** dye by AgNPs-**6**.

Temperature (K)	*k (min^{-1})*	*Ea (kJ mol^{-1})*	*ΔH (kJ mol^{-1})*	*ΔS (kJ mol^{-1}K^{-1})*	*ΔG (kJ mol^{-1})*
298	0.0223	40.81	38.19	-0.148	82.30
308	0.0363				83.78
318	0.7329				85.26
333	0.1242				87.48

S1.2.2. Suggested mechanism of photocatalytic dye degradation

It is primarily the reactive oxygen species (ROS) that include hydroxyl radicals (OH•), superoxide anion radicals (O_2─•), hydrogen peroxide (H_2O_2), and holes (h+) that deal with the degradation of organic pollutants [2]. Thus, in order to determine how various species contribute to deterioration, a study on radical scavenging was carried out. Four scavengers were used in the experiment: p-benzoquinone (p-BQ) for O_2─•, isopropanol (IPA) for OH•, L-ascorbic acid (L-AA) for H_2O_2, and disodium ethylenediamine acetate (Na_2EDTA) for H^+ [3].

The percent of dye degradation by all radical scavengers was decreased indicating that all reactive species were formed in the solution when illuminated by sunlight as indicated in Fig. 13B. Existence of p-BQ and Na_2EDTA however, decreased the degradation of dye. It indicates that the dye degradation took place on the catalyst with the significant role of O_2–• and H^+ generation. To gain a better insight of the electron degradation process and transport of electrons, valence band (VB) and conduction band (CB) potentials have been calculated with help of Butler-Ginley equations (Equation S5 and S6) [4].

$$E_{CB} = X - E_C - 0.5E_g \tag{S5}$$

$$E_{VB} = E_{CB} + E_g \tag{S6}$$

Where X, E_C and E_g indicate the Mulliken's absolute electronegativity (4.439 eV for Ag), energy of free electrons (4.5 eV), the bandgap energy determined from Tauc's plot (2.82 eV), respectively. The values of E_{CB} and E_{VB} were calculated to be -1.47 eV and 1.35 eV, respectively as described in Fig. **13C**. The band edge locations indicate that the formation of $O_2^{-•}$ is highly likely because the reduction potential of O_2/ $O_2^{-•}$ is −0.33 eV and the energy conduction band (E_{CB}) is at -1.138 eV. The results also supported the experimental findings of radical scavenging experiments because it revealed that in the presence of p-BQ, dyes degraded very little yet scavenging $O_2^{-•}$.

The performance of these radicals in the degradation phenomena, as shown in Equations S7–S15, discovered the degradation process of azo dyes. It was proposed that when the catalyst is exposed to sunlight, pairs of electrons (e^-) and holes (h^+) are created which in effect excite the valence electrons forming a conduction band. Reactive O_2–• is formed when the dissolved oxygen absorbs the electrons. Reactive oxygen species (ROS) are formed in the reaction of the produced radicals to different species of oxygen, which promotes the process of the degradation of dyes. Following the rest of the publications, a chronological process of the degradation of dye 7 by the photocatalytic heterogeneous reaction is offered [5].

i) The first step requires the activation of AgNPs-6 photo-catalyst through light energy absorbance by UV, visible or sunlight. This light energy must be at least as big, or bigger than the photo-catalyst band gap. This makes the electrons in the valence band excited and they are transferred to the conduction band leaving a space in the valence band. This process starts the degradation reaction, which can be seen in equation (S7).

$$AgNPs - 6 + hv\ (Uv) \rightarrow AgNPs - 6\ [h^+ (VB) + e^- (CB)] \qquad (S7)$$

ii) The conduction band electron then enters into a reduction reaction with dissolved oxygen to create a superoxide radical, as depicted in equation (S8):

$$O_2 + AgNPs - 6\ [e^- (CB)] \rightarrow O_2{}^- \qquad (S8)$$

iii) The hole in the valence band is where the oxidation of the water molecules oxidize and create hydroxyl radicals. Alternatively, the positively charged holes may neutralize cationic hydroxyl groups (see equation (S9)) to give $OH^•$ radicals:

$$H_2O_{(ads)} + AgNPs - 6\ [h^+ (VB)] \rightarrow HO^-{}_{(ads)} + H^+ \qquad (S9)$$

iv) The protons created in step iii) neutralize the superoxide radical, given in equation (S10):

$$O_2{}^-{}_{(ads)} + H^+ \rightleftarrows HOO^-{}_{(ads)} \qquad (S10)$$

v) The HOO^- radical produced in step iv) undergoes a reaction to yield hydrogen peroxide and O_2 gas, as shown in equation (S11):

$$2HOO^-{}_{(ads)} \rightarrow H_2O_2 + O_2{}_{(ads)} \qquad (S11)$$

vi) The decomposition of hydrogen peroxide formed in step v) results in the generation of hydroxyl free radicals, according to equation (S12):

$$H_2O_2{}_{(ads)} \rightarrow 2HO^-{}_{(ads)} \qquad (S12)$$

vii) The conduction band's electrons are reduced with the dye **7** in the process that leads to the creation of a reduced product, as shown by equation (S13):

$$Dye\ 7 + AgNPS - 6\,[e^-(CB)] \rightarrow Reduction\ products \qquad (S13)$$

viii) The holes of the valence band lead to oxidation of the dye 7 thereby forming the oxidation products, as depicted in equation (S14):

$$Dye\ 7 + AgNPs - 6\,[h^+(VB)] \rightarrow Oxidation\ products \qquad (S14)$$

ix) Hydroxide free radical reacts with MB dye to form several intermediate products, achieving the formation of carbon dioxide and water in the end of degradation, as described by equation (S15):

$$Dye\ 7 + 2HO^- \rightarrow intermediates \rightarrow CO_2 + H_2O \qquad (S15)$$

Figure 13C illustrates the mechanism of the AgNPs-**6** based photocatalytic degradation MB dye.

While the degradation rate of MB is competitive, the key advantage of the AgNPs-6 photocatalyst is its robust performance under natural sunlight and excellent reusability over multiple cycles. The visible-light activity is facilitated by the optimal bandgap, which is a direct consequence of the quantum confinement effect and the electronic influence of the indole-imine capping layer.

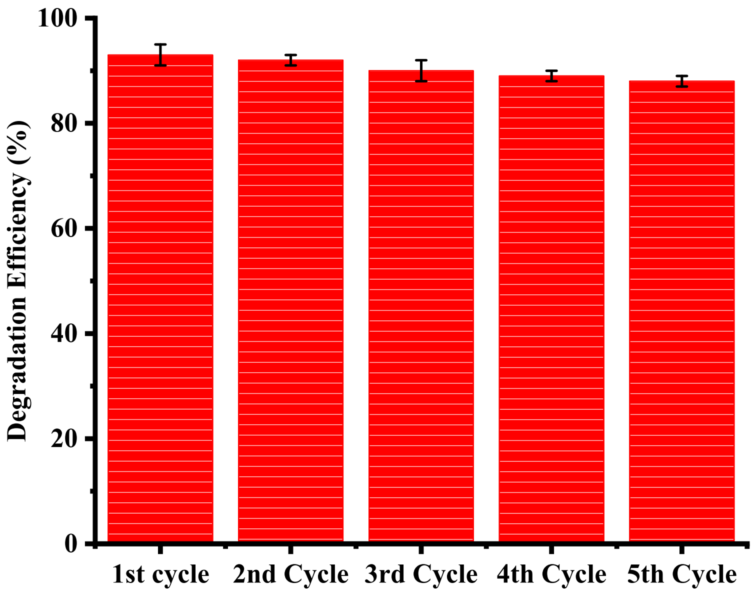

Fig. S6. Regeneration results showing the effectiveness of the AgNPs-**6** photocatalyst for dye **7** break down after five repeated operations and regeneration cycles.

DLS Report

Size Report - 2 Page Example

Malvern Panalytical

Sample Details

**Sample Name:**	8727 z.size Nasir Assad Uo Sargodha Sample 3 code i1		
**Project Name:**	Project 1		
**Date and Time:**	Tuesday, 6 January 2026 1:19:04 pm		
**Type:**	Size	**Result Source:**	Instrument
**Cell Name:**	DTS0012	**Temperature (°C):**	25
**Material Name:**	Polystyrene latex	**Dispersant Name:**	Water
**Material RI:**	1.59	**Dispersant RI:**	1.33
**Material Absorption:**	0.01	**Dispersant Viscosity (cP):**	0.887
		**Dispersant Dielectric Constant:**	78.5

Size Report - 2 Page Example

Malvern Panalytical

Size Distribution by Intensity

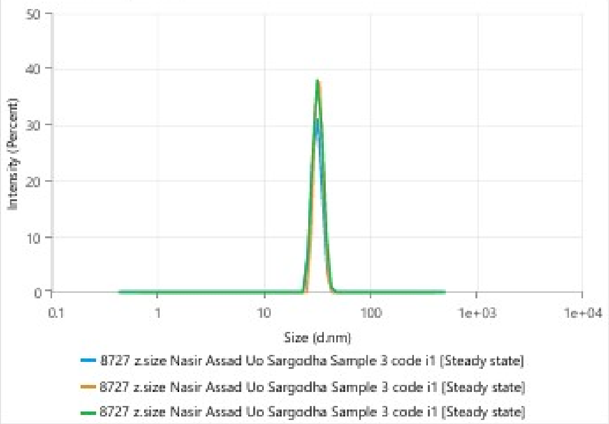

Size Distribution by Volume

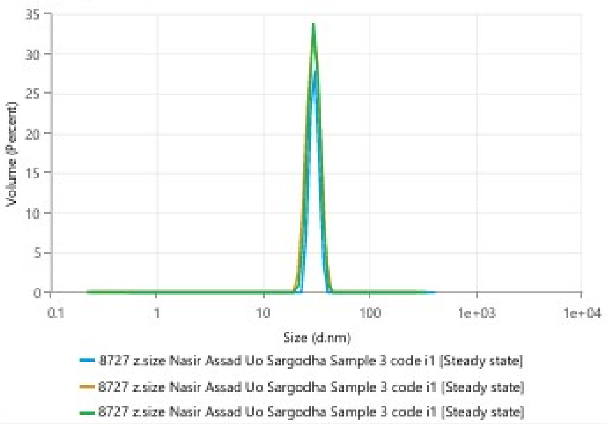

Size Report - 2 Page Example

Malvern Panalytical

Correlogram

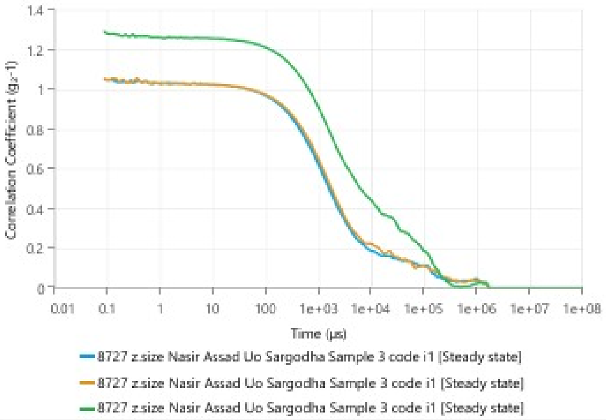

Cumulants Fit

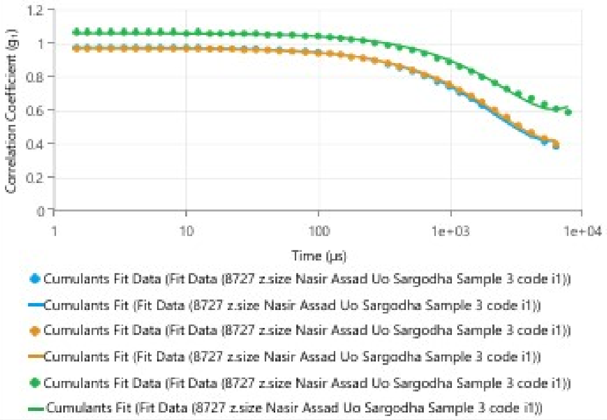

Size Report - 2 Page Example

Malvern Panalytical

Data Quality Guidance

8727 z.size Nasir Assad Uo Sargodha Samp
Larger-sized population

8727 z.size Nasir Assad Uo Sargodha Samp
Larger-sized population

8727 z.size Nasir Assad Uo Sargodha Samp
Number fluctuations

Statistics Table

Name	Mean	Standard Deviation	RSD	Minimum	Maximum
Z-Average (nm)	43.8	3.1	7.01	24.1	57.4
Polydispersity Index (PI)	0.6858	0.1671	24.37	0.583	0.8786
Intercept	0.9985	0.05198	5.206	0.9661	1.058
Fit Error	0.005419	0.002519	46.49	0.003181	0.008147
In Range (%)	78.02	1.648	2.113	76.24	79.49
Peak 1 Mean by Intensity ordered by area (nm)	36.2	2.5	6.91	33.8	49.3
Peak 2 Mean by Intensity ordered by area (nm)	0.00	-	-	0.00	0.00

Parameter List

Actual Instrument Settings\Instrument Serial Number : MAL1285161
Software Version : 3.2.0.84
Method Path :

ZS XPLORER

Malvern Panalytical Ltd.
www.malvernpanalytical.com

ZS XPLORER
Page 4 of 4

Template Created: 20/11/2019
Printed: 06/01/2026 1:57 pm

1. Khalid, Z., et al., *Causonis trifolia-based green synthesis of multifunctional silver nanoparticles for dual sensing of mercury and ferric ions, photocatalysis, and biomedical applications.* RSC advances, 2025. **15**(21): p. 16879-16893.
2. Zhou, R., et al., *Regulation of excitons dissociation in AgI/Bi3O4Br for advanced reactive oxygen species generation towards photodegradation.* Applied Catalysis B: Environmental, 2021. **285**: p. 119820.
3. Li, M.-r., et al., *Phototransformation of zinc oxide nanoparticles and coexisting pollutant: Role of reactive oxygen species.* Science of The Total Environment, 2020. **728**: p. 138335.
4. Ashraf, M.A., et al., *Fabrication of silver phosphate-ilmenite nanocomposites supported on glycol chitosan for visible light-driven degradation, and antimicrobial activities.* International journal of biological macromolecules, 2021. **169**: p. 436-442.

5. Khan, A.W., et al., *Sunlight-assisted green synthesis of gold nanocubes using horsetail leaf extract: A highly selective colorimetric sensor for Pb2+, photocatalytic and antimicrobial agent.* Journal of Environmental Chemical Engineering, 2024. **12**(3): p. 112576.
